# Intron Lariat RNA Inhibits MicroRNA Biogenesis by Sequestering the Dicing Complex in *Arabidopsis*

**DOI:** 10.1371/journal.pgen.1006422

**Published:** 2016-11-21

**Authors:** Ziwei Li, Shengpeng Wang, Jinping Cheng, Chuanbin Su, Songxiao Zhong, Qi Liu, Yuda Fang, Yao Yu, Hong Lv, Yun Zheng, Binglian Zheng

**Affiliations:** 1 State Key Laboratory of Genetic Engineering, Collaborative Innovation Center for Genetics and Development, School of Life Sciences, Fudan University, Shanghai, China; 2 Faculty of Life Science and Technology, Kunming University of Science and Technology, Kunming, China; 3 National Key Laboratory of Plant Molecular Genetics, Shanghai Institute of Plant Physiology and Ecology, Chinese Academy of Sciences, Shanghai, China; 4 Yunnan Key Laboratory of Primate Biomedical Research, Institute of Primate Translational Medicine, Kunming University of Science and Technology, Kunming, China; Gregor Mendel Institute of Molecular Plant Biology, AUSTRIA

## Abstract

Lariat RNAs formed as by-products of splicing are quickly degraded by the RNA debranching enzyme 1 (DBR1), leading to their turnover. Null *dbr1* mutants in both animals and plants are embryo lethal, but the mechanism underlying the lethality remains unclear. Here we characterized a weak mutant allele of *DBR1* in *Arabidopsis*, *dbr1-2*, and showed that a global increase in lariat RNAs was unexpectedly accompanied by a genome-wide reduction in miRNA accumulation. The *dbr1-2* mutation had no effects on expression of miRNA biogenesis genes or primary miRNAs (pri-miRNAs), but the association of pri-miRNAs with the DCL1/HYL1 dicing complex was impaired. Lariat RNAs were associated with the DCL1/HYL1 dicing complex *in vivo* and competitively inhibited the binding of HYL1 with pri-miRNA. Consistent with the impacts of lariat RNAs on miRNA biogenesis, over-expression of lariat RNAs reduced miRNA accumulation. Lariat RNAs localized in nuclear bodies, and partially co-localize with HYL1, and both DCL1 and HYL1 were mis-localized in *dbr1-2*. Together with our findings that nearly four hundred lariat RNAs exist in wild type plants and that these lariat RNAs also associate with the DCL1/HYL1 dicing complex *in vivo*, we thus propose that lariat RNAs, as decoys, inhibit miRNA processing, suggesting a hitherto unknown layer of regulation in miRNA biogenesis.

## Author Summary

It is known that lariat RNAs formed during pre-mRNA splicing are debranched by DBR1 (RNA debranching enzyme 1). Loss of function of *DBR1* causes embryo lethality in both animals and plants. In animals, some debranched lariat RNAs could be further processed into mirtron miRNAs, a class of nonconventional miRNAs that bypass the microprocessor for their biogenesis. However, no mirtron has been functionally validated in plants, and how the accumulation of lariat RNA in *dbr1* results in embryo lethality remains unclear. Here, we show that *DBR1* is necessary for the regulation of genome-wide miRNA biogenesis in plants. By investigating the correlation between lariat RNA accumulation and miRNA processing, we showed that the DBR1-mediated lariat RNA debranching process provides a safeguard role for the binding of the dicing complex with miRNA precursors. As both the DBR1-mediated lariat RNA debranching process and miRNA biogenesis are common features in higher eukaryotes, the finding that lariat RNAs sequester the dicing complex in plants may have a broad implications for the non-coding RNA field.

## Introduction

In higher eukaryotes, splicing of mRNA precursors (pre-mRNA), a critical step for gene expression, comprises two catalytic steps [1]. In the first step, the 5’ splice site is cleaved and concurrently the 5’ end of the intron is joined to the branch nucleotide by forming a phosphodiester bond. This results in the production of a 5’ exon and a 3’exon-containing lariat precursor. In the second step, the lariat precursor is cleaved at the 3’ splice site and the two exons are ligated to produce the mRNA. The excised intronic lariat RNA is linearized for degradation by an RNA debranching enzyme (DBR1) [2].

DBR1 was originally identified from budding yeast (*Saccharomyces cerevisiae*) in a study aimed at identifying host cellular factors involved in transposition of the Ty1 retrotransposon [3]. The *dbr1*Δ mutant of *S*. *cerevisiae* has reduced Ty1 transposition frequency and also shows increased accumulation of lariat RNAs. DBR1 is not essential for cell viability in *S*. *cerevisiae*. However, the *dbr1*Δ mutant in fission yeast (*Saccharomyces pombe*) exhibits severe growth defects [2]. Moreover, both the *dbr1* mutant in *Arabidopsis* [4] and mice [5] are embryo lethal. DBR1 is a single copy gene in eukaryotes [6], and homologues of DBR1 isolated from plants or animals can complement the *S*. *pombe dbr1* mutant [7], indicating that DBR1 function is conserved.

MicroRNAs (miRNAs) are a class of 21-24nt small RNAs generated from endogenous stem-loop transcripts. miRNA biogenesis begins with the processing of primary miRNAs (pri-miRNAs), which contain a hairpin structure that is cleaved twice by the dicing complex, which is mainly composed of the nuclear RNase III enzyme Dicer-Like 1 (DCL1) [8] and an RNA binding protein HYL1 [9], yielding mature miRNAs. DCL1 and HYL1, together with pri-miRNAs, are co-localized in nuclear dicing bodies [10,11], and HYL1 facilitates the binding of DCL1 to enhance the efficiency and accuracy of miRNA processing [12,13].

Previous findings showed that DBR1 is required for mirtron miRNA biogenesis in animals [14, 15], where it is independent of cleavage by the microprocessor. The existence of mirtron miRNAs in embryos might contribute the embryo lethality of the null *dbr1* mutant in animals [14]. However, no mirtron miRNA has been functionally validated in plants [16]. In addition, earlier studies showed that DBR1 is required for intronic snoRNAs biogenesis in yeast [17, 18], but plant snoRNAs are originated from either an intronic or a non-intronic context [19, 20], indicating that the involvement of DBR1 in snoRNA biogenesis is not sufficient to explain the important role of DBR1 during embryo development. Here, we report that lariat RNAs inhibit global miRNA biogenesis in *Arabidopsis*, possibly explaining the detrimental effects of lariat over-accumulation. We provide evidence that the over-accumulation of lariat RNAs caused genome-wide reduction of miRNAs in *Arabidopsis*, and show that lariat RNAs associated with the DCL1/HYL1 dicing complex to compete with pri-miRNAs. Moreover, we demonstrated that hundreds of lariat RNAs exist in wild type plants, suggesting that these lariat RNAs might be biologically relevant. Given the observations of lariat RNAs in embryonic stem cells [21] and evidence for lariat RNAs acting as decoys of RNA binding proteins [22], we thus propose that lariat RNAs function as decoys of the dicing complex to maintain a proper processing of pri-miRNAs.

## Results

### DBR1 is required for miRNA accumulation in *Arabidopsis*

**Fig 1:**
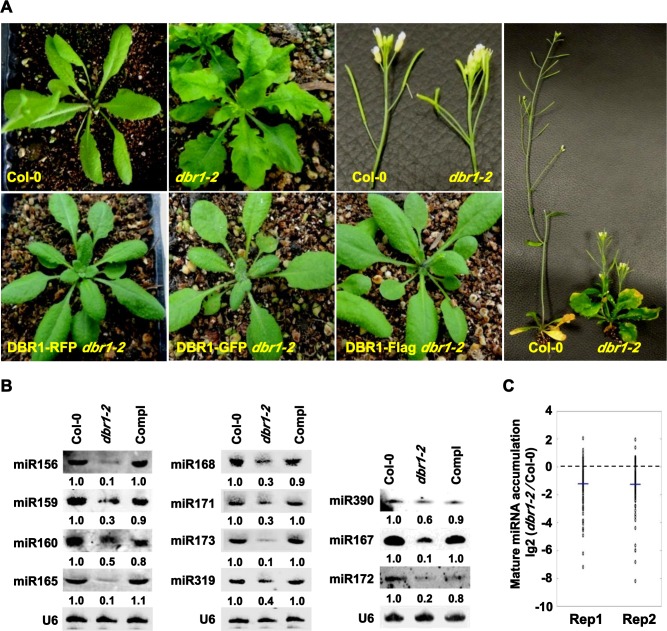
DBR1 is required for miRNA accumulation. (A) Morphological phenotypes of Col-0 and *dbr1-2* plants. DBR1-GFP *dbr1-2*, DBR1-RFP *dbr1-2*, and DBR1-Flag *dbr1-2* represent rescued lines of *dbr1-2* with different protein fusion constructs driven by the native *DBR1* promoter. (B) miRNA northern blot analysis in Col-0, *dbr1-2*, and a *dbr1-2* transgenic line containing the *pDBR1*::*DBR1-RFP* transgene (Compl). The numbers indicate the relative abundance of miRNAs among the three genotypes and represent the mean of three repeats (P < 0.05). U6 was used as a loading control. (C) Deep sequencing analysis of miRNAs in Col-0 and *dbr1-2*. Small RNA libraries were generated from inflorescences of two biological replicates. The normalized abundances of miRNAs were calculated as reads per ten million (RPTM), and log2-transformed ratios of *dbr1-2*/Col-0 were plotted. Each circle represents one miRNA. Thick lines indicate mean values. Rep1 and Rep2 denote replicate 1 and replicate 2, respectively.

In an ethyl methanesulfonate mutagenesis screen aimed at isolating mutants compromised in miRNA biogenesis using *dcl1-14* as a parental line [23], we isolated a mutant with pleiotropic developmental phenotypes, which included curly and serrated leaves, increased branching, short stature, and reduced fertility (Fig 1A). Whole genome sequencing identified a G-to-A mutation in the coding region of *DBR1*, which encodes an RNA debranching enzyme (*DBR1*) (S1A and S1B Fig). This mutation caused the conversion of a glycine, located within the LRL motif required for substrate binding (S1A and S1B Fig) [24], a region highly conserved in DBR1 in *S*. *pombe*, animals, and plants, to an arginine (S1A and S1B Fig). When *DBR1* genomic fragments driven by the native promoter and fused to GFP, RFP, or Flag, respectively, were introduced into this mutant, the phenotypes were completely rescued (Fig 1A). We identified a T-DNA insertion allele in *DBR1* only in a heterozygous state and crossed it to the homozygous mutant isolated from our genetic screen. Approximately half (83/178) of the F1 plants exhibited mutant phenotypes (S1C Fig). Therefore, the mutant is an allele of *DBR1*. Both a previously isolated loss-of-function allele of *DBR1* [4] (here renamed *dbr1-1*) and the T-DNA insertion line (here named *dbr1-3*) are embryo lethal. The abundance of DBR1 was unaffected in our weak mutant allele (here named *dbr1-2*) (S1D Fig).

Considering that the *dbr1-2* mutant resembles mutants that are defective in miRNA accumulation, we examined miRNA accumulation in *dbr1-2* by northern blot analysis. Compared to those in Col-0, the levels of tested miRNAs were reduced in *dbr1-2* (Fig 1B). pDBR1::DBR1-RFP (Compl) fully restored the levels of these miRNAs (Fig 1B). To determine whether DBR1 is required for global miRNA accumulation, we compared mature miRNA levels in *dbr1-2* and Col-0 by small RNA deep sequencing analysis. Results from two replicates confirmed a genome-wide reduction of miRNA levels in *dbr1-2* (Fig 1C and S1 Table). Compared to many canonical miRNAs reduced in *hyl1* [25, 26], our northern blot assay (Fig 1B) and deep sequencing analysis (S1 Table) showed that most HYL1-dependent miRNAs from 32 canonical miRNA families, such as miR156, miR159, and miR160, were also obviously reduced in *dbr1-2* (S1 Table), indicating that DBR1 and HYL1 have overlapping functions in miRNA biogenesis. Due to the potential feedback regulation between lariat RNA debranching and pre-mRNA splicing, we wondered whether the effects of DBR1 on miRNA biogenesis was dependent on the possible function of DBR1 on splicing. Moreover, recent studies showed that many *MIR* genes contain introns and those *MIR* genes with introns usually stimulate biogenesis of miRNAs originating from such intron-containing precursors [27, 28]. By analyzing the genomic structure of 54 *MIR* genes with reduced miRNAs in *dbr1-2*, we showed that only 8 *MIR* genes contain introns (S1 Table), and that among more than 15 intronic miRNAs in *Arabidopsis*, only 4 intronic miRNAs were reduced in *dbr1-2* (S1 Table), suggesting that the involvement of DBR1 in miRNA biogenesis might be unrelated to the possible function of DBR1 in pre-mRNA splicing, and thus that the effects of DBR1 on miRNA biogenesis are independent of the properties of *MIR* genes. We thus concluded that DBR1 is required for miRNA accumulation in plants.

### Pri-miRNAs over-accumulate in the *dbr1-2* mutant

To exclude the possibility that the reduced miRNA levels observed in *dbr1-2* were due to altered expression of the miRNA pathway components, we performed quantitative RT-PCR (qRT-PCR) to determine transcript levels of genes that have been shown to act in miRNA biogenesis [8, 9, 29, 30]. As shown in S2A Fig, the expression levels of these genes were comparable to those in Col-0; Western blot analysis further confirmed that the protein levels of those key components in the miRNA pathway in *dbr1-2* were comparable to those in Col-0 (S2B Fig). These results indicate that the *dbr1-2* mutation had no effects on the expression of the miRNA pathway components.

**Fig 2:**
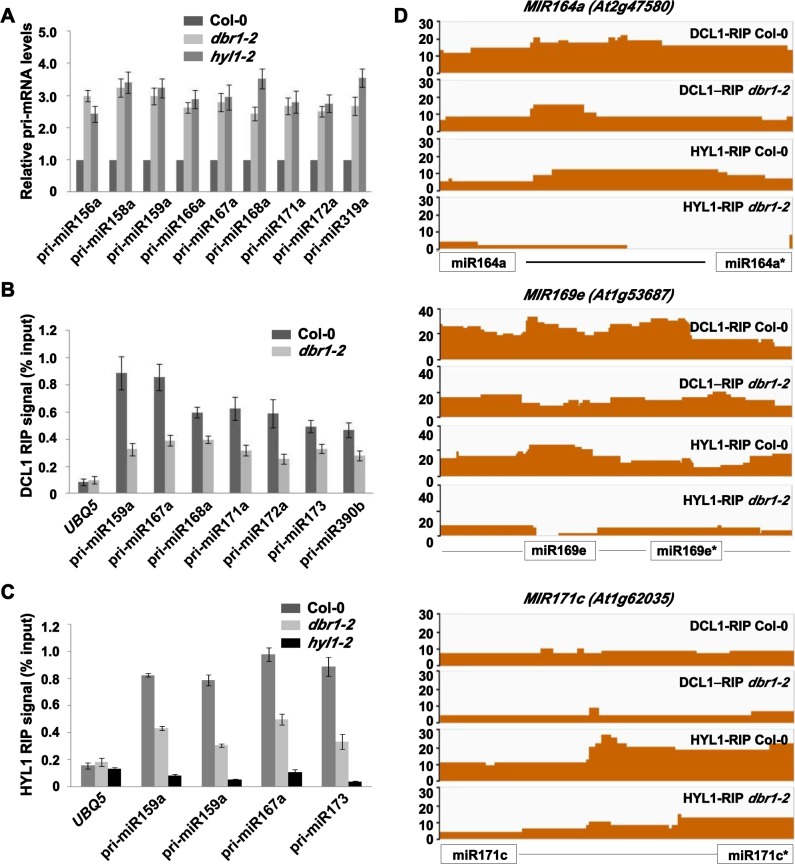
Pri-miRNA binding to DCL1 and HYL1 is reduced in *dbr1-2*. (A) Detection of pri-miRNAs in Col-0, *dbr1-2* and *hyl1-2* by qRT-PCR. *UBQ5* was the loading control. Standard deviations were calculated from three biological replicates. (B and C) Association between pri-miRNAs and the DCL1/HYL1 complex by RIP analysis. RNA was immunoprecipitated from inflorescences or seedlings of Col-0 and *dbr1-2* using DCL1 or HYL1 antibodies, respectively. The amount of pri-miRNAs was determined by qRT-PCR and normalized to the input. *hyl1-2* was used as the negative control for HYL1 antibody. *UBQ5* was used as a negative control. Error bars show SE calculated from three biological replicates. (D) The occupancy of DCL1 and HYL1 at *MIR164a*, *MIR169e*, and *MIR171c*. The coverage regions are shown as normalized peaks. The *x* axis indicates the relative position of miRNA and miRNA* location. The *y* axis indicates normalized peaks from the genomic region. Reads counts were normalized to tag per 10 million (TP10M) to adjust for sequencing depth differences of the two RIP-seq libraries.

The reduced miRNA accumulation in *dbr1-2* could be caused by reduced pri-miRNA levels. To test whether it was the case, we examined the levels of pri-miRNAs in Col-0 and *dbr1-2* plants. qRT-PCR analyses showed that the levels of the nine pri-miRNAs tested were increased by 2- to 4-fold in *dbr1-2* (Fig 2A), a fold-change similar to that found in *hyl1* (Fig 2A). The observation of increased levels of pri-miRNA prompted us to examine whether DBR1 plays a role in the transcription of miRNA genes (*MIR*). Pol II is responsible for *MIR* transcription in animals and plants [31–33]. Therefore, we first examined Pol II occupancy at promoters of *MIR* genes by chromatin immunoprecipitation (ChIP), using an antibody against the second largest subunit of Pol II (NRPB2). Compared to the no antibody control (S2C Fig), Pol II occupancy at *MIR* was comparable to that in *dbr1-2* (S2D Fig). To further exclude the possibility that the over-accumulation of pri-miRNAs in *dbr1-2* was due to increased transcription of *MIR*, we examined the effect of *dbr1-2* on the expression of a *GUS* reporter gene driven by either the *MIR172a* promoter (*MIR172a*::*GUS*) or the *MIR390b* promoter (*MIR390b*::*GUS*). The transgenic lines *MIR172a*::*GUS* and *MIR390b*::*GUS* were separately crossed with *dbr1-2* and progeny plants homozygous for both transgene and *dbr1-2* were analyzed. Both GUS activity (S2E Fig) and the levels of *GUS* transcripts (S2F Fig) in *dbr1-2* were comparable to those in Col-0, while the levels of pri-miR172a and pri-miR390b were increased in the *dbr1-2* lines (S2F Fig). Taken together, these results indicate that DBR1 is not required for *MIR* transcription.

### The binding of pri-miRNA with the dicing complex is impaired in *dbr1*-2

That pri-miRNAs over-accumulated and the levels of mature miRNAs were reduced in *dbr1-2* suggested that miRNA processing might be impaired. We then investigated whether the association of pri-miRNAs with the dicing complex was compromised in *dbr1-2* using RNA immunoprecipitation (RIP) experiments. We performed RIP assays, using DCL1 antibody, with Col-0 and *dbr1-2* inflorescences, and using HYL1 antibody with Col-0, *dbr1-2*, and *hyl1-2* seedlings. Pri-miRNAs were detected by qRT-PCR from immunoprecipitated RNAs. The amounts of the pri-miRNAs bound by both DCL1 (Fig 2B) and HYL1 (Fig 2C) were significantly reduced in the *dbr1-2* background. To determine whether DBR1 is required for genome-wide pri-miRNA binding with the dicing complex, we compared the levels of DCL1- or HYL1-immunoprecipitated miRNA precursors (pri-miRNA) from Col-0 and *dbr1-2* by RNA sequencing analysis. We found that most reads were predominantly mapped to the whole region of *MIR* genes. However because the boundaries of most pri-miRNAs are not clear, it was not feasible to calculate the abundance of pri-miRNAs, and thus we only counted reads uniquely mapped to pre-miRNA regions. We found that pre-miRNAs were overall enriched in both DCL1- and HYL1-immunoprecipitated samples in Col-0 (Fig 2D and S2 Table). Furthermore, enrichment of most pre-miRNAs with detectable RIP-seq abundances (more than 5 RPKM in either the *dbr1-2* or Col-0 libraries), in both DCL1- and HYL1-immunoprecipitated samples, were reduced in *dbr1-2* (Fig 2D and S2 Table). These results indicate that the association of pri-miRNAs with the dicing complex was disrupted in *dbr1-2*, which leads to reduced miRNA processing.

### Characterization of lariat RNAs in *Arabidopsis*

Because DBR1 strongly binds to lariat RNAs [34], we hypothesized that DBR1 might directly act in the binding of pri-miRNAs with the dicing complex. To test this hypothesis, we performed a RIP assay using DBR1 antibodies, but found that DBR1 was not associated with pri-miRNA *in vivo* (S3A Fig). However, DBR1 is obviously associated with lariat RNAs (S3A Fig), which is consistent with previous studies [34]. In addition, a Co-IP assay showed no association between DBR1 and the miRNA biogenesis machinery (S3B Fig). These results therefore make it unlikely that DBR1 itself is a component of the dicing complex during miRNA processing.

**Fig 3:**
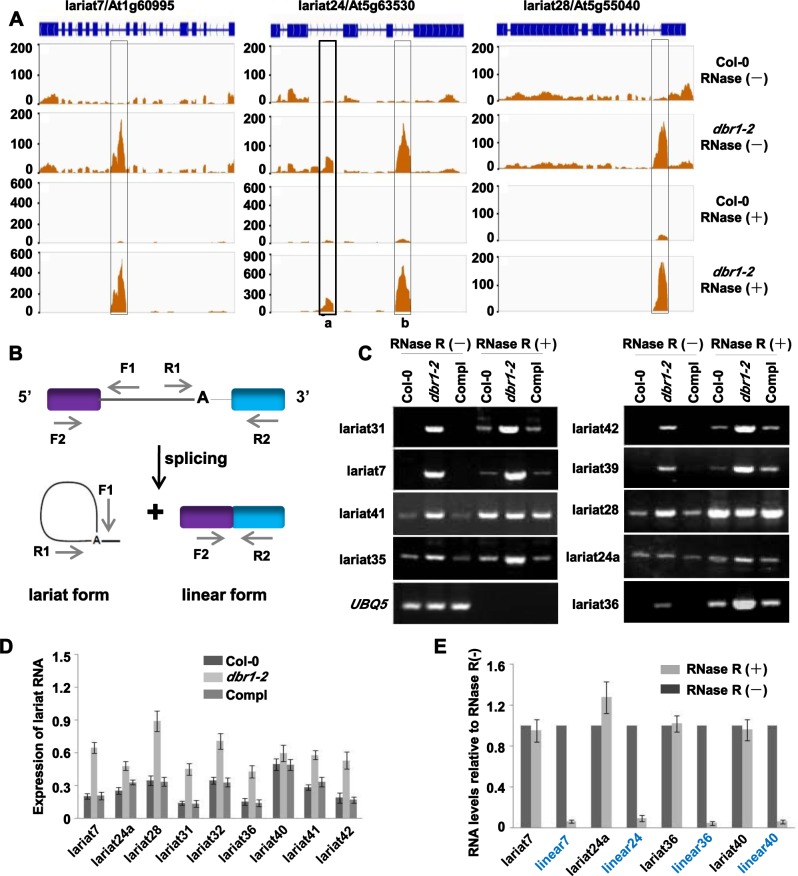
Characterization of lariat RNAs in plants. (A) Three examples of identified lariat RNAs. Normalized peaks of lariat RNAs are highlighted by black rectangles. The exon is boxed in blue, and the intron is a line. The X-axis indicates the chromosomal location. The Y-axis indicates normalized peaks from the genomic region. Reads counts were normalized to tag per 10 million (TP10M) to adjust for differences in sequencing depth of the two RNA-seq libraries. (B) Schematic of divergent primers for lariat RNAs and convergent primers for linear mRNAs. The purple and blue boxes indicate exons; A represents the branch point. (C) Validation of lariat RNAs by RT-PCR with the divergent PCR primer pairs shown in (B) in Col-0, *dbr1-2*, and Compl (pDBR1::DBR1-RFP *dbr1-2*) with or without RNase R treatment. *UBQ5* was used as the loading control. (D) Validation of lariat RNAs by qRT-PCR using total RNA of Col-0, *dbr1-2*, and Compl (pDBR1::DBR1-RFP *dbr1-2*). The amount of lariat RNAs was normalized to *UBQ5*. Error bars show SE calculated from three biological replicates. (E) qRT-PCR showing resistance of lariat RNAs to RNase R digestion. Linear mRNAs (blue font indicated) are positive controls for RNase R treatments. The amount of RNAs after RNase R treatment was normalized to the RNase R-untreated sample. Error bars show SE calculated from three biological replicates.

Loss-of-function of *DBR1* causes global accumulation of lariat RNAs in yeast [2]. To investigate the relationship between the accumulation of lariat RNAs and miRNA biogenesis, we tested genome-wide how many lariat RNAs accumulate in *dbr1-2*. As RNase R specifically degrades linear RNAs, while keeping the loop portion of a lariat RNA intact [35], our strategy was to globally compare total RNAs in *dbr1-2* and Col-0, with or without RNase R-treatments. Therefore, we performed RNA-seq after constructing libraries of ribosomal RNA (rRNA)-depleted RNAs, with or without RNase R-treatments (S3C Fig). By selecting uniquely mapped intronic reads, 1560 intronic RNAs were identified as potential lariat RNAs in *dbr1-2* (Fig 3A and S3 Table). After RNase R treatment, most of them (1534) were still detectable (Fig 3A and S3 Table), indicating that these lariat RNAs exist as stable circular forms. Unexpectedly, approximately 23% (360/1534) of these lariat RNAs also exhibited significant expression (> = 5 RPKM) in wild type plants (S3 Table), suggesting that these lariat RNAs naturally escaped debranching, a phenomenon recently shown in human cells [21].

To validate lariat RNAs, we performed RT-PCR using sets of divergent primers (Fig 3B). As shown in Fig 3C and 3D, most tested lariat RNAs obviously over-accumulated in *dbr1-2*. Some lariat RNAs, such as lariat24a, lariat28, lariat35, and lariat41, were easily detected in Col-0 (Fig 3C), further confirming that these lariat RNAs naturally escaped the debranching activity of DBR1. Further qRT-PCR analyses of RNase R-untreated samples confirmed the results of RT-PCR (Fig 3D). To test whether the lariat RNAs were circular, we performed RT-PCR using RNase R-treated RNAs as templates, and showed that all tested lariat RNAs were circular (Fig 3C). To determine whether RNase R treatments were complete, we performed qRT-PCR analyses using both RNase R-untreated and RNase R-treated samples, and showed that lariat RNAs were stably detectable in both samples, while the corresponding linear mRNAs were almost eliminated by RNase R treatments (Fig 3E). Sanger sequencing further confirmed that lariat RNAs are circular (S3D Fig). Taken together, these results suggest that both lariat RNAs over-accumulated in *dbr1-2*, and lariat RNAs naturally present in wild type plants, might play certain roles in biological processes.

### Lariat RNAs affect the binding of the DCL1/HYL1 complex with pri-miRNAs

**Fig 4:**
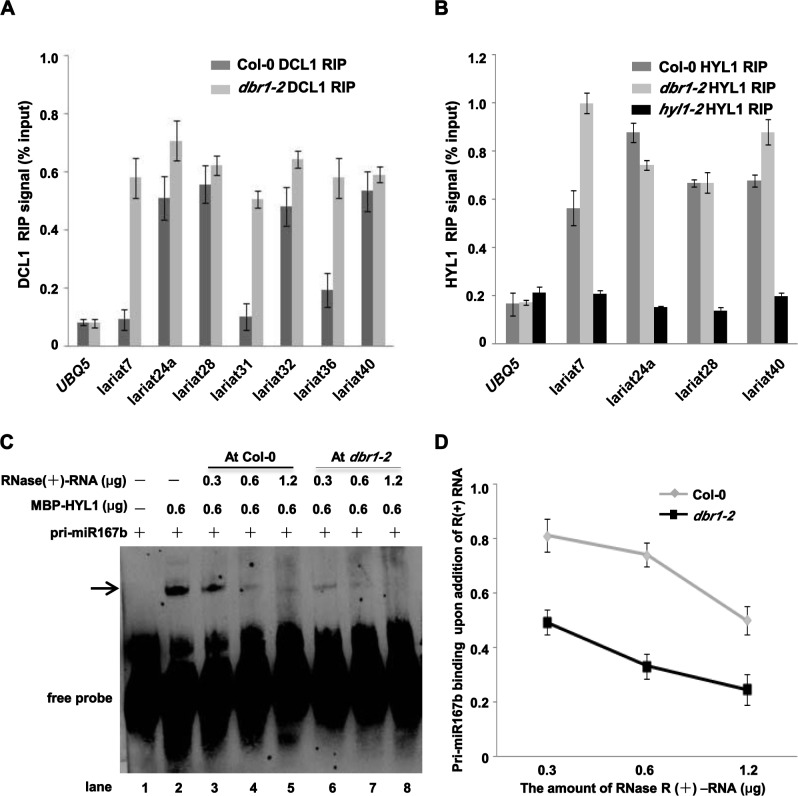
Lariat RNAs competitively inhibit DCL1/HYL1 binding to pri-miRNAs. (A) Lariat RNAs associate with DCL1 using a RIP assay performed as in Fig 2B. Immunoprecipitated RNAs were analyzed by qRT-PCR with divergent primers to detect the indicated lariat RNAs. *UBQ5* was used as the loading and negative control. Error bars show SE calculated from three biological replicates. (B) Lariat RNAs associate with HYL1 using a RIP assay performed as in Fig 2C. qRT-PCR was performed according to (A). *hyl1-2* was used as the negative control. *UBQ5* was used as the loading and negative control. Error bars show SE calculated from three biological replicates. (C) R-EMSA to determine HYL1 binding to *pri-miR167b* in the presence of circular RNAs from Col-0 or *dbr1-2* plants. Recombinant MBP-HYL1-D1D2 (MBP-HYL1) was incubated with a 5’_biotin_labeled *pri-miR167b* probe after addition of different amounts of circular RNAs isolated from Col-0 or *dbr1*-2 inflorescences, respectively. The arrow indicates the HYL1-*pri-miR167b* complex. (D) Hybridization intensities were quantified and normalized to the controls (lane 2 in C), and are shown in the line graph. Bars represent the average normalized intensity of three biological replicates.

To investigate whether lariat RNAs accumulated in *dbr1-2* were correlated with reduced binding of the dicing complex to pri-miRNAs, we first investigated whether these lariat RNAs were associated with the DCL1/HYL1 complex *in vivo*. Lariat RNAs were present in both DCL1- and HYL1-immunoprecipitates in *dbr1-2* (Fig 4A and 4B). Notably, compared to that of negative control, *UBQ5*, and other lariat RNAs hardly detected in Col-0 (lariat7, lariat31, lariat36), several tested lariat RNAs naturally present in Col-0, such as lariat24a, lariat28, lariat32, and lariat40, were also bound by both DCL1 and HYL1 (Fig 4A and 4B), indicating that lariat RNAs that naturally escaped debranching could be bound by the DCL1/HYL1 dicing complex.

Due to the observations that lariat RNAs are circular and that most circular RNAs accumulated in *dbr1-2* could be lariat-derived, we then investigated whether the association of the DCL1/HYL1 complex with lariat RNAs affected binding of the DCL1/HYL1 complex to pri-miRNAs. We performed RNA electrophoretic mobility shift assays (R-EMSA) with Col-0 and *dbr1-2*, to compare the binding capacity of recombinant HYL1 (S4A Fig) with biotin-labeled *pri-miR167b* in the presence or absence of lariat RNAs. Consistent with a previous study [36], HYL1 specifically bound *pri-miR167b* as indicated by the arrow (S4B Fig), while other unrelated recombinant proteins MBP and GST (S4A Fig) did not bind *pri-miR167b* (S4B Fig). Notably, DBR1 recombinant protein showed no binding with *pri-miR167b* (S4B Fig), further supporting that DBR1 itself is not directly required for the binding of pri-miRNA with the dicing complex. To investigate whether lariat RNAs regulate HYL1 binding with pri-miRNA, we performed competition assays. Notably, due to the limitations of synthesizing lariat RNA *in vitro* in our conditions, and because lariat RNAs should be the most abundant population of circular RNAs in *dbr1* mutants, we used RNase R-digested RNAs (R(+)-RNA) to perform the competition assays. Increasing amounts (0.3 to 1.2 μg) of cold R(+)-RNA were added to the binding reaction mixture containing HYL1 and labeled *pri-miR167b*. As shown in Fig 4C and 4D, the signal corresponding to the HYL1-*pri-miR167b* complex was decreased proportionally to the amount of cold circular RNA added from *dbr1-2*. Unexpectedly, the addition of cold circular RNA from Col-0 in the binding reaction also produced moderate competition effects (Fig 4C and 4D), while linear single-stranded RNA of *GAPDH* had minor competition effects on HYL1 binding (S4C Fig).

Considering that the function of DBR1 is highly conserved in eukaryotes, we speculated that lariat RNAs from other species might play similar roles in binding the dicing complex. To test this idea, we examined the effects of circular RNAs from a fission yeast *dbr1*Δ strain on the binding capacity of HYL1 with *pri-miR167b*. Compared to the binding in the control (S4D Fig), circular RNAs from the yeast *dbr1* mutant greatly attenuated the binding of HYL1 (S4D and S4E Fig). Taken together, these results suggest that circular RNAs, most likely lariat RNAs, could play a regulatory role in binding of the dicing complex to pri-miRNAs.

### Over-expression of lariat RNAs causes reduced miRNA accumulation

**Fig 5:**
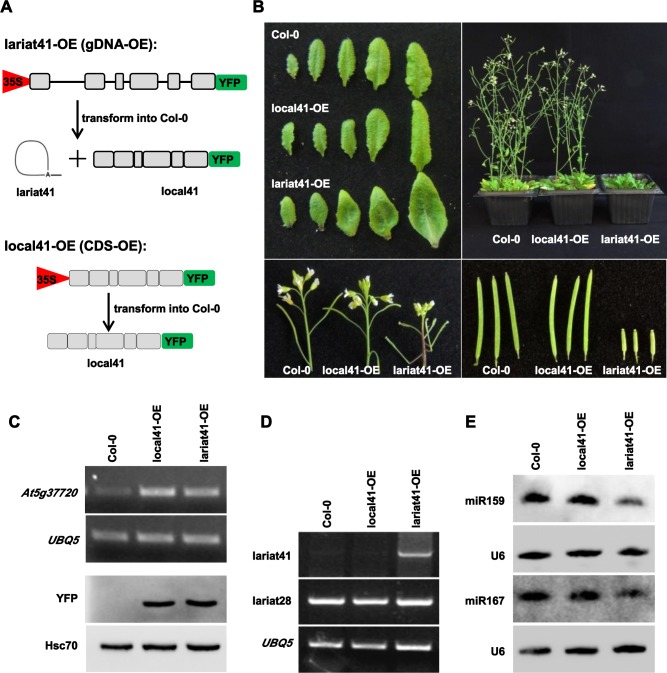
Over-expression of lariat41 caused reduced miRNA accumulation. **(A)** Strategy to over-express lariat41 in *Arabidopsis*. Full length genomic sequence (indicated as lariat41-OE/gDNA-OE) or full length coding sequence (indicated as local41-OE/CDS-OE) was driven by the 35S promoter and fused by an YFP tag, and introduced into plants. Grey boxes indicate exons, and lines indicate the intron. (B) Morphological phenotypes of transgenic plants over-expressing lariat 41 (lariat41-OE) and the corresponding gene of lariat41 (local41-OE). Curly leaves, late flowering, altered phyllotaxy, and reduced fertility were shown in lariat41-OE. More than 10 independent transgenic lines were obtained for each. (C) RT-PCR and western blot analysis to detect expression of the corresponding gene of lariat41. Total RNA from inflorescences of a representative T3 transgenic line for each was used for cDNA synthesis, and At5g37720 was amplified to indicate the mRNA level of the corresponding gene, *UBQ5* as the loading control. In similar, YFP was detected using total protein from inflorescences by western blot, Hsc70 as the loading control. Two additional biological replicates were performed, and similar results were obtained. (D) RT-PCR to detect expression of lariats. Total RNA from inflorescences of a representative T3 transgenic line for each was used for cDNA synthesis, and lariat41 was amplified, lariat28 was used as the negative control, and *UBQ5* as the loading control. Two additional biological replicates were performed, and similar results were obtained. (E) miR159 and miR167 northern blot analysis in Col-0, local41-OE, and lariat41-OE. U6 was used as a loading control. Another biological replicate was performed, and similar results were obtained.

Since it seems that lariat RNAs compete for HYL1/DCL1 binding to pri-miRNAs, thus reducing their processing and then the accumulation of miRNAs, we hypothesized that any method that changed lariat RNAs levels would have an impact on miRNA accumulation. To test this, we generated over-expression (OE) lines of lariat RNA and assessed miRNA accumulation. Although how some lariat RNAs escape debranching in wild type plants remains unknown, we hypothesized that over-expressing the corresponding genomic DNA should lead to increased levels of intron-derived lariat RNAs. Here, we selected lariat41, which is detectable in wild type plants (Fig 3C and S3 Table), to investigate whether over-expression of lariat RNA would affect miRNA accumulation. We generated more than 10 independent transgenic plants over-expressing the genomic DNA of At5g37720 (lariat41-OE) or the CDS of At5g37720 (local41-OE) (Fig 5A). Only lariat41-OE transgenic plants exhibited pleiotropic phenotypes (Fig 5B), which were reminiscent of mutants deficient in miRNA accumulation. Both RT-PCR and western blot analysis showed comparable mRNA and protein levels of At5g37720 in local41-OE and lariat41-OE plants (Fig 5C). As expected, lariat41 was significantly increased in lariat41-OE lines but not in local41-OE lines (Fig 5C), while lariat28, an unrelated lariat RNA, was equal among the three genotypes (Fig 5D). Importantly, we showed that levels of miR159 and miR167 were reduced in lariat41-OE but not in local41-OE plants (Fig 5E), indicating that increased lariat41 levels were anti-correlated with accumulation of miRNAs. To further support this anti-correlation, we transiently expressed lariat42 in tobacco leaves (S5 Fig) with a split YFP separated by lariat42-originated intron sequences (S5A Fig). The YFP signal was detected, indicating that the lariat42-originated intron was properly spliced (S5B Fig). RT-PCR analysis showed that lariat42 accumulated in the infiltrated leaves but not in control leaves (S5C Fig). Northern blot analysis showed that miR167 was reduced in leaves over-expressing lariat42 (lariat42-OE). Taken together, these results indicate that lariat RNA accumulation is negatively correlated with miRNA levels.

### Lariat RNAs are recruited into nuclear bodies and partially co-localize with HYL1

**Fig 6:**
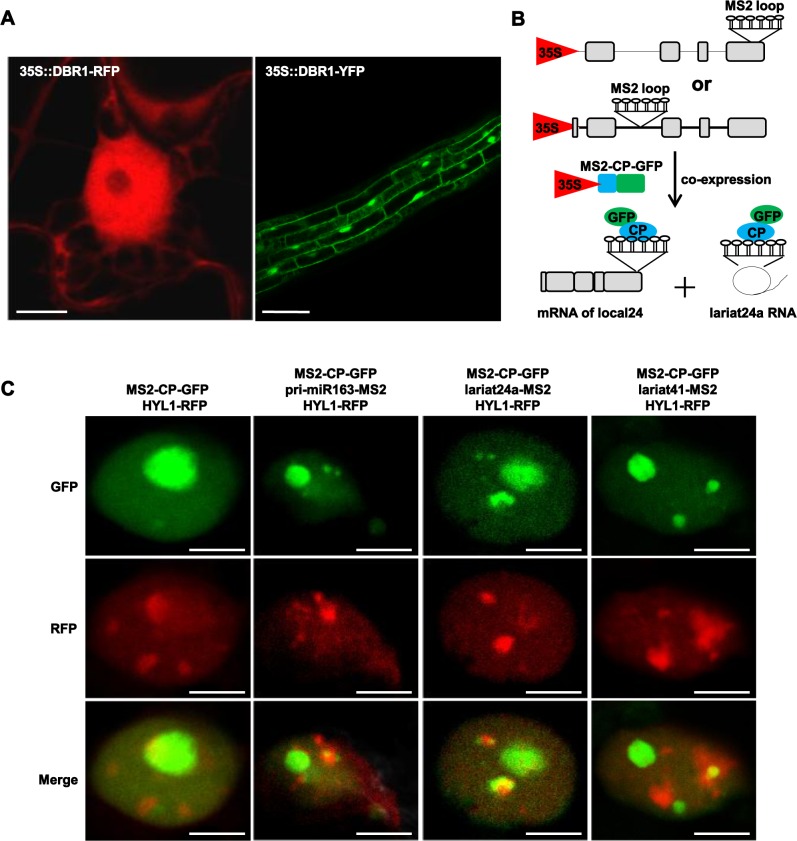
Subcellular localization of DBR1 and lariat RNA. (A) Subcellular localization of *Arabidopsis* DBR1. 35S::DBR1-RFP was transiently expression in tobacco leaves and RFP signal was observed after 48 hr. Roots from 35S::DBR1-GFP transgenic plants were observed under the GFP channel. (B) Strategy to visualize lariat RNAs in live cells. The MS2 sequence (indicated as stem-loops) was inserted into a lariat24a-located intron. A co-expressed GFP-tagged MS2-CP protein was used to visualize lariat RNAs. Grey boxes indicate exons, and lines indicate the intron. (C) Genomic DNA of lariat24a and lariat41 was fused to 6XMS2 repeats and co-infiltrated into tobacco leaves with MS2-CP-GFP and HYL1-RFP. The genomic DNA of pri-miR163 was used as the positive control. Scale bars = 10 μm.

Pre-mRNA splicing occurs in the nucleus and lariat RNAs are byproducts of splicing, so we reasoned that DBR1 might localize in the nucleus. Transient expression of DBR1-RFP in tobacco as well as transgenic *Arabidopsis* plants expressing DBR1-GFP showed that DBR1 was distributed in both the nucleus and cytoplasm (Fig 6A), which is consistent with the finding that DBR1 is a nucleocytoplasmic shuttling protein in humans [37]. The DBR1-2 mutant protein did not affect the distribution of DBR1 (S6A Fig).

Because processing of pri-miRNAs occurs in the nucleus, we hypothesized that nuclear lariat RNAs might be co-localized with components of the DCL1/HYL1 complex. To test this idea, we modified the MS2 system [38] to visualize endogenous lariat RNAs in plants (Fig 6B and S6B Fig). Six copies of the binding site for the RNA-binding MS2 coat proteins (MS2-CP) were inserted into the lariat24a- or lariat41-generating intronic regions of At5g63530 (Fig 6B) and At5g37720 (S6B Fig), respectively. Expression of MS2-CP fused to GFP carrying an NLS signal (MS2-CP-GFP) enables the visualization of lariat24a and lariat41 tagged with MS2-binding sites. We co-transformed tobacco with different combinations of plasmids harboring MS2-CP-GFP, HYL1-RFP or lariat24a-MS2 and lariat41-MS2 under the control of the 35S promoter. To differentiate the subcellular localization of lariat RNA and the mRNA of the corresponding gene, we co-transformed a plasmid harboring the full length genomic DNA fused to 6X MS2 at the end of the last exon of the lariat24a corresponding gene, under control of the 35S promoter. As a positive control for dicing bodies, we co-transformed a plasmid harboring pri-miR163-MS2 under control of the 35S promoter. We analyzed the subcellular localization of MS2-CP-GFP by fluorescence microscopy. MS2-CP-GFP uniformly accumulated in one big dot in the nucleus (Fig 6C), while HYL1-RFP accumulated in small dots in addition to the big dot in the nucleus in some nuclei (Fig 6C). Notably, in many nuclei HYL1-RFP was also distributed more evenly without any dots (S6C Fig), a localization consistent with a previous study [39]. As expected, when the plasmid of pri-miR163-MS2 was introduced, we observed small bodies surrounding the big dot in some nuclei (Fig 6C), and these nuclear bodies were partially co-localized with HYL1-nuclear bodies (Fig 6C), consistent with previous localization studies [10, 11]. Intriguingly, lariat24a and lariat41 also accumulated in small nuclear bodies surrounding the big dot in some nuclei (Fig 6C and S6D and S6E Fig), and similar to pri-miR163, both lariat24a and lariat41 partially co-localized with HYL1-nuclear bodies (Fig 6C and S6D and S6E Fig). As a negative control, we showed that the mRNA of the lariat24a corresponding gene was uniformly distributed in all examined nuclei (S6C Fig). Collectively, our data indicate that lariat RNAs are partially recruited into nuclear bodies.

### Localization of the dicing complex in the nucleus was impaired in *dbr1-2*

**Fig 7:**
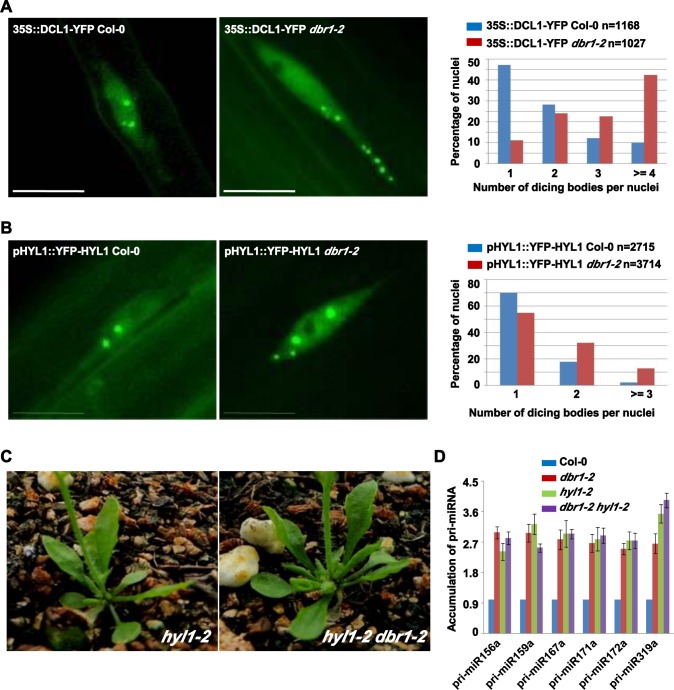
DCL1 and HYL1 are mis-localized in *dbr1-2* mutants. (A-B) Subnuclear localization of DCL1 (A) and HYL1 (B) in Col-0 and *dbr1-2* root cells. Right panels show the percentage distribution of dicing bodies per cell. The X-axis represents the number of dicing bodies per cell, and the Y-axis represents the percentage of cells with the corresponding numbers. “n” represents the numbers of analyzed cells. (C) Morphological phenotypes of the *hyl1-2* and the *dbr1-2 hyl1-2* double mutant. Pictures were taken of 5-week-old plants. (D) Expression levels of indicated pri-miRNAs in Col-0, *dbr1-2*, *hyl1-2*, and *dbr1-2 hyl1-2* plants by qRT-PCR. *UBQ5* was used as an internal control and for normalization of the data. Standard deviations were calculated from three technical replicates. The results shown were reproduced with three biological replicates. Scale bars = 10 μm.

Several mutants deficient in miRNA biogenesis exhibit abnormal patterns of dicing bodies [40–42], which might reflect a deficiency in the function of the dicing complex. To examine whether DBR1 affects localization of the dicing complex, we examined the effects of *dbr1-2* on the sub-nuclear localization of DCL1 and HYL1. Progeny homozygous for both transgenes (DCL1-YFP and YFP-HYL1) and *dbr1-2* were obtained. In Col-0, more than 85% of 1168 root cells harbored three or fewer dicing bodies in the nucleus and less than 10% of root cells harbored more than three dicing bodies (Fig 7A). In contrast, *dbr1-2* had significantly more DCL1-containing nuclear bodies (Fig 7A); more than 40% of 1027 root cells contained four or more dicing bodies (Fig 7A). A similar observation was made for HYL1-containing dicing bodies (Fig 7B). Taken together, these results suggest that DBR1 is required for the proper sub-nuclear localization of the dicing complex.

To further dissect the genetic relationship between lariat RNA debranching and pri-miRNA processing, we crossed *dbr1-2* to *hyl1-2*. The double mutant was morphologically similar to *hyl1-2* (Fig 7C), and expression levels of pri-miRNAs (Fig 7D) in *hyl1-2 dbr1-2* were comparable to that in *hyl1-2*, suggesting that DBR1 and HYL1 act in miRNA biogenesis in the same genetic pathway. Taken together, these observations indicate the debranching process of lariat RNAs is tightly coupled with pri-miRNA processing, and thus that non-debranched lariat RNAs are conveniently accessible for the dicing complex to balance the binding of pri-miRNAs.

## Discussion

Although a few plant miRNAs originate from intronic regions, it is becoming increasingly apparent that miRNA biogenesis has to be coordinately regulated by pre-mRNA splicing, since many mutants of genes involved in splicing exhibit defects in miRNA biogenesis [29, 43–45]. However, the mechanism by which pre-mRNA splicing affects miRNA biogenesis remains largely unexplored. Here we demonstrated that lariat RNAs, as so-called by-products of pre-mRNA splicing, play a negative role in regulation of miRNA homeostasis. Lariat RNAs act as a molecular sponge to sequester the dicing complex, and thus maintain a steady level of mature miRNAs. Therefore, our data provide new insights into how by-products of pre-mRNA splicing, other than the previously thought spliceosome itself, play a role in miRNA biogenesis.

**Fig 8:**
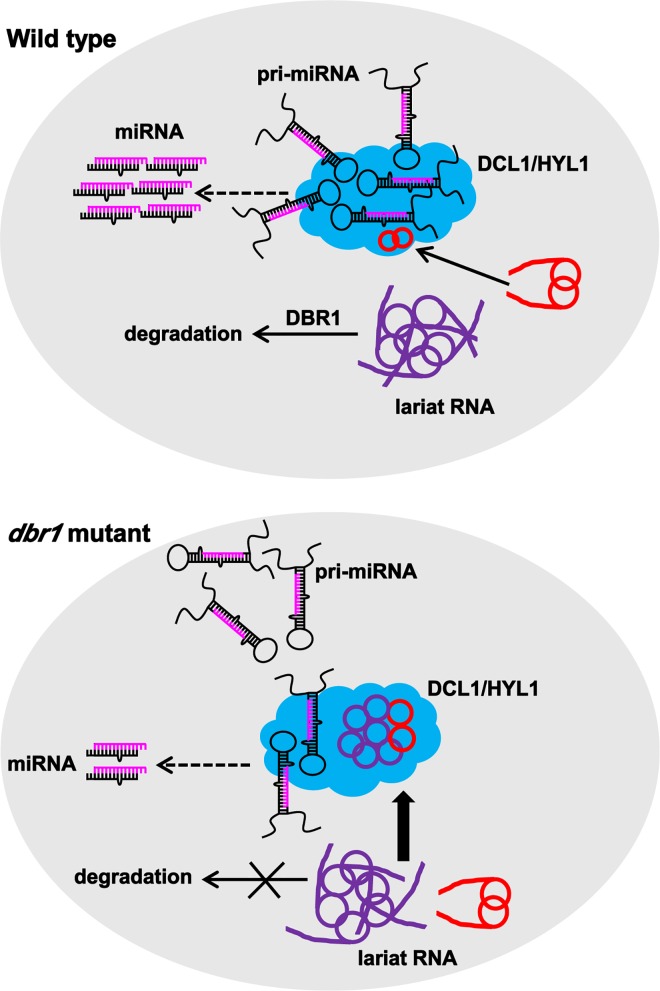
A Model for lariat RNA inhibits miRNA biogenesis in plants. Pri-miRNAs are specifically recruited to the DCL1/HYL1 complex (blue indicated) for processing. In wild type plants, most lariat RNAs (indicated in purple) are quickly debranched by DBR1, only a small portion of lariat RNAs (indicated in red) naturally escapes debranching, also associates with the DCL1/HYL1 complex. In contrast, in *dbr1-2*, over-accumulated lariat RNAs might be recognized by the DCL1/HYL1 complex, and thus attenuate the association between the DCL1/HYL1 complex and pri-miRNAs, producing less miRNA.

Our results suggest that the debranching process of lariat RNAs contributes to the proper processing of pri-miRNA by balancing the binding of pri-miRNA to the DCL1/HYL1 complex. As we proposed, in wild type plants (Fig 8, upper panel), DBR1 is able to degrade most lariat RNAs, but some lariat RNAs naturally escape debranching, as also shown in human embryonic stem cells [21]. In *Arabidopsis*, we showed that these non-debranched lariat-derived circular RNAs act as a molecular sponge of the dicing complex, in which lariats play a potential competitive role to avoid excessive pri-miRNA processing by attracting the DCL1/HYL1 complex. In contrast, in *dbr1-2* (Fig 8, lower panel), the over-accumulation of lariat RNAs disrupts the binding of the DCL1/HYL1 complex to pri-miRNA, and thus causes less miRNA production. In conclusion, our results uncover a new layer of miRNA biogenesis regulation by other RNA molecules, and establish the link between the splicing process and the generation of small RNAs that had not been conceived previously.

It is well known that pri-miRNA binding to the dicing complex is an important step in miRNA biogenesis, but how the dicing complex differentiates pri-miRNAs from other structurally related RNAs is unclear. A previous study in plants showed that SINE RNAs, potentially with loop structures, compete with pri-miRNAs for HYL1 binding [46], and a study in human cells showed that the microprocessor recognizes the basal UG and apical UGU motifs of pri-miRNAs [47]. Interestingly, lariat RNAs contain the GU/UG motif from the 5’ splicing site next to the branch point, indicating that the GU/UG motif might be wrongly recognized by the DCL1/HYL1 complex in plants. In addition, recent studies in animals showed that DGCR8 binds other RNAs besides pri-miRNAs [48], and that Dicer globally binds to many loop RNAs [49], which thus sequesters Dicer to reduce miRNA accumulation [49], suggesting that binding of the miRNA biogenesis machinery could be regulated.

Indeed, together with the findings that lariat RNA acts as sources for both mirtron miRNA biogenesis in animals [14, 15] and siRNA biogenesis in yeast [50], our finding that lariat RNA inhibits global miRNA processing in plants implicates a widespread involvement of lariat RNA in small RNA biogenesis. In species such as yeast, which lack miRNAs, lariat RNAs are further processed into siRNA for gene silencing [50]. In contrast, in eukaryotes such as *Drosophila* and *Arabidopsis*, which are enriched in miRNAs, lariat RNA might have switched its role from siRNA biogenesis to miRNA biogenesis. Elucidation of additional genetic modifiers of lariat RNA binding and processing should provide more insights into how lariat RNA acts in small RNA biogenesis.

## Materials and Methods

### Plant materials, yeast strains, and antibodies

*Arabidopsis thaliana* Columbia (Col-0) is wild type. Seeds of *dbr1-3* (SALK_047099), *hyl1-2* (SALK_064863), and MIR390b::GUS (CS66477) were obtained from the Arabidopsis Biological Resources Center (ABRC, www.arabidopsis.org). 35S::DCL1-YFP seeds were generated in our laboratory. pHYL1::YFP-HYL1 seeds were obtained from Yuda Fang’s lab. Wild-type and *dbr1* fission yeast strains were purchased from Bioneer (http://pombe.bioneer.co.kr). Anti-DCL1 (Agrisera, #AS122102), anti-HYL1 (Agrisera, #AS06136), anti-SE (Agrisera, #AS09532), anti-Hsc70 (Stressgen, SPA-018), anti-GFP (Covance, #MMS-118R), and anti-FLAG (Sigma, #F7425) antibodies were purchased. Anti-DBR1, anti-NRPB2, and anti-AGO1 antibodies were generated by our laboratory.

### Plasmid construction

For construction of the DBR1-GFP, DBR1-RFP, DBR1-Flag, 35S::DBR1-RFP, and 35S::DBR1-YFP plasmids containing the endogenous promoter, *DBR1* was amplified from Col-0 genomic DNA with the primer pair DBR1F1/R1, cloned into pENTR-D/TOPO, and then transferred into the plant expression destination vector pMDC107 to construct DBR1-GFP, into pMDC163 (GUS was replaced by mRFP) to construct DBR1-RFP, into pEarleyGate302 to construct DBR1-Flag, into pEarleyGate101 (HA-GFP was replaced by mRFP) to construct 35S::DBR1-RFP, and into pB7YWG2 to construct 35S::DBR1-YFP. To construct 35S::DBR1-Flag, the *DBR1* CDS was obtained using the primer pair DBR1F2/R2 and cloned into the plant expression vector pCambia1306. To construct 35S::DBR1-2-YFP, the CDS sequences of *DBR1* was amplified from *dbr1-2* with primer pairs DBR1F2/R2 and cloned into pCambia2302. To construct DBR1-GST, the CDS sequences of *DBR1* was amplified with primer pairs DBR1F3/R2 and cloned into pGEX2TK. To construct DCL1-YFP, HYL1-RFP, and HYL1-Flag, the CDS sequences of *DCL1* and *HYL1*, respectively, were obtained using the primer pairs DCL1F1/R1 and HYL1F1/R1, cloned into pENTR-D/TOPO, and then transferred into the plant expression destination vectors pEarleyGate101, pEarleyGate101 (YFP-HA was replaced by mRFP), and pEarleyGate202, respectively. To construct SE-Flag, the CDS sequence of *SE* was obtained using the primer pair SEF1/R1, and cloned into the plant expression vector pCambia1306. To construct lariat41-OE, the full length genomic region of At5g37720 was amplified using primer pair lariat41F2/R2 and ligated to pENTR1A, and transferred into pB7YWG2. To construct local41-OE, the full length coding region of At5g37720 was amplified from Col-0 cDNA using primer pair lariat41F2/R2 and ligated to pENTR1A, and transferred into pB7YWG2. To construct lariat42-OE, the intron sequences of At5g43100 were amplified using primer pair lariat42F2/R2 and inserted between nYFP and cYFP, and transferred into pB7WG2. To transfer lariat24a and lariat41 into the MS2 system, we first constructed lariat24a-pENTR-D/TOPO and lariat41-pENTR1A by introducing full length genomic fragments of At5g63530 and At5g37720 into pENTR-D/TOPO and pENTR1A using the primer pairs gL24F/R and gL41F/R, respectively, and then the 6XMS2 loops were amplified with primers 6XMS2Loops_F1/R1 and 6XMS2Loops_F2/R2 from 35S::GW-6XMS2, then ligated into lariat24a-pENTR-D/TOPO by XmnI and lariat41-pENTR1A by SacI, respectively, then transferred into the plant expression vector pB7WG2. To construct the mRNA control of At5g63530 into the MS2 system (local24-MS2), the plasmid of lariat24a-pENTR-D/TOPO were introduced into 35S::GW-6XMS2 by LR reaction. To construct pri-miR163 into the MS2 system, we first constructed pri-miR163-pENTR-D/TOPO by introducing full length genomic fragments of *MIR163* into pENTR-D/TOPO with the primer pair miR163-MS2-F/R, and then transferred into the plant expression vector 35S::GW-6XMS2. Primer sequences are listed in S4 Table.

### Small RNA sequencing analysis

Illumina sequencing of small RNA libraries from inflorescences was performed by Genergy (Shanghai, China), and small RNA libraries were constructed according to Illumina's instructions using a TruSeq Small RNA Library Preparation kit. The small RNA sequencing profiles were analyzed as described [51]. Briefly, the sequencing reads were filtered to remove reads with more than 5 nucleotides with sequencing scores smaller than 20. Adaptor sequences were removed to extract small RNAs. Redundant sequences were eliminated, and unique small RNAs were counted. Unique small RNAs were mapped back to the *A*. *thaliana* reference genome using SOAP2 [52]; mature miRNAs and pre-miRNAs (downloaded from miRBase, http://www.mirbase.org/, v19) were found using BLASTN. Then, custom programs were used to calculate the normalized abundance (RPTM, Reads Per Ten Million sequencing tags) of mature miRNAs. miRNAs with at least 100 RPTM in either Col-0 or *dbr1-2* were used to calculate log2 fold changes of their RPTM values. Deep sequencing datasets were deposited in the SRA database of National Center for Biotechnology Information with accession No. SRP062035.

### miRNA northern blot

Total RNA was extracted using Trizol reagent from inflorescences. 5 μg small RNAs enriched by PEG8000 were separated by denaturing 15% (w/v) PAGE and transferred to a nylon membrane. 5’_biotin_labeled-oligo nucleotide sequences complementary to miRNA were synthesized as probes. Hybridization was performed using hybridization buffer (Ambion), and signals were detected using chemiluminescent nucleic acid detection module. U6 was used as a loading control. The probes are listed in S4 Table.

### R-EMSA

R-EMSA was performed according to [36] with modifications. Biotin-labeled *pri-miR167b* was synthesized by *in vitro* transcription. Using an RNA EMSA Kit (Thermo Scientific), electrophoresis mobility shift assay experiments were performed in a 20 μl reaction system containing binding buffer, 5% (v/v) glycerol, 2 μg tRNA, 2 nM of biotin-labeled *pri-miR67b* transcripts, and purified MBP-HYL1_D1D2 (only two DsRBD domains). For the competition assay, total RNA treated by RNase R was added to the reaction at different concentrations. The reaction was incubated for 30 min, electrophoresed on a 6.5% (w/v) native PAGE gel, and transferred to a nylon membrane, then detected using a chemiluminescent nucleic acid detection module.

### RIP (RNA IP)

Chromatin from inflorescences of Col-0 and *dbr1-2* was immunoprecipitated with anti-DCL1 and anti-HYL1 antibodies. For the DBR1 RIP assay, chromatin from inflorescences of Col-0 was immunoprecipitated with anti-DBR1 antibodies. RNA recovered from the immunoprecipitates was used for cDNA synthesis with Oligo dT. The primer sets used for PCR are listed in the S4 Table. For RIP-seq analysis, recovered RNAs were used for library preparation with Illumina TruSeq Stranded Total RNA HT Sample Prep Kit (P/N15031048), and then subjected to deep sequencing with Illumina HiSeq 2000 at Genergy (Shanghai, China). The RIP-seq libraries were aligned to the genome with Cufflinks 2 [53]. The "bedtools genomecov" command of bedtools [51] was used to calculate genome coverage of RIP-seq libraries. Then, a custom program was used to calculate the RPKMs (Reads Per Kilo basepairs and per Million sequencing tags) of pre-miRNAs in miRBase (v21), using the genome coverage of RIP-seq libraries. Deep sequencing datasets were deposited in the SRA database of National Center for Biotechnology Information with accession No. SRP063916.

### ChIP (Chromatin Immunoprecipitation)

ChIP was performed similar to RIP, the difference is that DNA not RNA is recovered. Pol II occupancy at miRNA loci was determined by ChIP using anti-RPB2 antibody and 2-week-old seedlings from Col-0 and *dbr1-2*, respectively. Immunoprecipitated DNA was quantified by qPCR relative to total input DNA. The results shown were consistent in three biological replicates. The primer sets used for the PCR are listed in S4 Table.

### GUS Staining

Three week-old seedlings from transgenic plants in Col-0 and *dbr1-2* backgrounds was fixed in 90% acetone for 2–3 h and then stained for 12 h in 50 mM sodium phosphate buffer, pH 7.0, containing 0.2% Triton X-100, 5 mM potassium ferrocyanide, 5mM potassium ferricyanide, and 1 mM X-Gluc, then washed in 70% ethanol three times. Images were taken with a Leica DFC295 stereoscope.

### Pipeline for lariat RNA annotation by RNA-seq analysis

Total RNA isolated from Col-0 and *dbr1-2* was first treated with a Ribo Zero kit (Epicenter) to obtain a ribosomal RNA-depleted RNA (ribo^-^ RNA). Then ribo^-^ RNA was incubated with or without RNase R (Epicentre) and then subjected to phenol:chloroform purification. Purified RNAs were further used for library preparation with Illumina TruSeq Stranded Total RNA Sample Prep Kit, and then subjected to deep sequencing with Illumina HiSeq 2000 at Genergy (Shanghai, China). Stranded RNA-seq reads were mapped to the *Arabidopsis* genome annotation (TAIR 10) with Cufflinks 2 [53]. CuffDiff in Cufflinks 2 was used to find deregulated genes. Genes with at least 5 RPKM in either *dbr1-2* or Col-0 and *q*-values smaller than 0.05 were designated deregulated genes. The "bedtools genomecov" command of bedtools [53] was used to calculate the genome coverage of RNA-seq libraries. Then, a custom program was used to calculate the RPKMs (Reads Per Kilo basepairs and per Million sequencing tags) of introns of annotated genes in TAIR10, using the genome coverage of RNA-seq libraries. Introns that had at least 5 RPKM in *dbr1-2* profiles and had multiple test corrected *P*-values of smaller than 0.05, calculated with edgeR [54], were designated as enriched introns. Deep sequencing datasets were deposited in the SRA database of National Center for Biotechnology Information with accession No. SRP062035.

### Detection of lariat RNAs

Lariat RNAs across the branch site were detected by RT-PCR as described [35]. Total RNA with or without RNase R-treatment were used as templates. cDNA synthesis was carried out using SuperScript III (Invitrogen) with random hexamers. The reaction mixtures were incubated at 30°C for 10 min, at 42°C for 120 min, at 50°C for 30 min, at 60°C for 30 min, and at 99°C for 5 min. We then used divergent primer sets to detect lariat RNAs by PCR or qPCR. Primer sequences used are listed in S4 Table.

### Recombinant protein expression and purification

The corresponding plasmids of DBR1-pGEX2TK and HYL1 (D1D2)-pMAL were introduced into the expression host BL21-CodonPlus (DE3). Cells was grown to an OD600 of 0.4–0.6, and expression of GST or MBP fused proteins were induced by addition of 0.1 mM IPTG and incubation at 16°C for overnight. Cells carrying the pGEX2TK or pMAL empty vectors were also grown and induced as controls. Cell pellets were resuspended in lysis buffer and purified according to the recommended protocols of Glutathione Sepharose4B resin (for GST) or the amylose resin (for MBP), respectively.

### Transient expression in *N*. *benthamiana* and co-immunoprecipitation

35S::DCL1-YFP with 35S::DBR1-Flag, 35S::HYL1-Flag, and 35S::SE-Flag, respectively, were co-expressed in *N*. *benthamiana*. Leaves were ground in liquid nitrogen and homogenized in lysis buffer (50 mM Tris_HCl (pH 8.0), 150 mM NaCl, 0.2% Nonidet P-40, 2 mM DTT, 5% glycerol, proteinase inhibitor) and centrifuged for 15 min at 13200 rpm. The lysate was incubated with GFP-Trap agarose beads (Chromotek) for 2 h. The immune complexes were then washed with lysis buffer. Proteins retained on the beads were resolved on SDS-PAGE. Anti-GFP and anti-Flag antibodies were used to detect DCL1, and DBR1, HYL1 or SE, respectively, by using western blot analysis.

### Data deposition

The data reported in this paper have been deposited in the SRA database of National Center for Biotechnology Information with accession No. SRP062035 for small RNA seq and RNA-seq, and accession No. SRP063916 for RIP-seq.

## Supporting Information

S1 Fig DBR1 is required for the regulation of miRNA activity. (A) Partial amino acid sequence alignment of DBR1 from various species. DBR1 from Mouse (*Mus musculus*, NP_113580.2), Human (*Homo sapiens*, NP_057300.2), Fish (*Danio rerio*, NP_955947.1), Plant (*Arabidopsis thaliana*, NP_567881.1), Fly (*Drosophila melanogaster*, NP_996022.1), Worm (*Caenorhabditis elegans*, *NP_491868*.*2)*, and fission yeast (*Schizosaccharomyces pombe*, NP_593470.2), were aligned using ClustalW. Identical residues are highlighted in yellow. Asterisk indicates the Gly that was mutated to Arg (G135R) in *dbr1-2*. (B) The protein structure of DBR1. The closed triangle indicates the T-DNA insertion site in *dbr1-1 and dbr1-3*. The arrow indicates the location of the mutation of glycine to arginine in *dbr1-2*. MPE indicates the metallophosphoesterase domain, LRL indicates the lariat recognition loop, and CTD indicates a novel C-terminal domain. (C) Allelic analysis of *dbr1-2* and *dbr1-3*. A small field was shown for 3-week-old growing F1 progeny, and *dbr1-2*-like plants are highlighted by red circles. (D) Western blot to determine DBR1 levels in wild type (Col-0) and *dbr1-2* using anti-DBR1 antibody. # indicates the endogenous DBR1 in Col-0 and *dbr1-2*, and * indicates DBR1-Flag and DBR1-GFP in 35S::DBR1-Flag or 35S::DBR1-GFP transgenic plants, respectively. Notably, the loading amount in Col-0 and *dbr1-2* is half of that of two transgenic plants. Hsc70 served as a loading control.
(TIF)

S2 Fig DBR1 has no effects on expression of miRNA genes and *MIR* transcription. (A) RT-qPCR analysis of mRNA levels of miRNA biogenesis genes. SE was calculated from three biological replicates. (B) Western blot to determine levels of DCL1, HYL1, SE, AGO1, and NRPB2 in Col-0 and *dbr1-2*. Hsc70 was the loading control. (C) and (D) ChIP performed with Col-0 (dark gray bars) or *dbr1-2* (light gray bars) with anti-NRPB2 antibody. SE was calculated from three biological replicates. (E) Representative GUS staining images of MIR172a::GUS and MIR390b::GUS transgenic plants in Col-0 and *dbr1-2* backgrounds. (F) RT-qPCR analysis of the transcript levels of *GUS*, *pri-miR172a*, and *pri-miR390b* in the indicated plants. SE was calculated from three biological replicates.
(TIF)

S3 Fig DBR1 is not associated with DCL1 and DBR1 does not bind *pri-miR167b*. (A) RIP performed with *dbr1-2* inflorescences using anti-DBR1 antibody (light gray bars) or no antibody (dark gray bars) as the negative control. RNA was immunoprecipitated from inflorescences of *dbr1-2*. The amount of pri-miRNAs was determined by RT-qPCR and normalized to the input. *UBQ5* was used as a negative control. Lariat RNAs served as positive controls. Error bars show SE calculated from three biological replicates. (B) Co-immunoprecipitation between DCL1 and DBR1, SE, and HYL1. DCL1-YFP was co-expressed with DBR1-Flag, SE-Flag, or HYL1-Flag in *N*. *benthamiana*. HYL1-Flag and SE-Flag were used as positive controls. (C) Schematic flow of RNA-seq analysis. 5 μg total RNA were digested by Dnase I, and then RNA were removed ribosomal RNA using Ribo-Zero kit. The half amount of RNAs were treated by RNase R as R(+) RNA to remove linear RNAs, the other half amount of RNAs were used as R(-) RNA. Those RNA fragments with size larger than 100 nt were used for library construction and sequencing. (D) Chromas figures of partial sequences from lariat7 and lariat24a by Sanger sequencing. PCR products of lariat7 and lariat24a from Fig 3C were sequenced using the primer lariat7R and lariat24aR, respectively. The asterisk represents branch points “A” in the DNA sequence, here it was reversed to “T” after cDNA synthesis. The “GT” downstream of the branch point was indicated the 5’ splice site. The 5’ intron sequence means the partial intron sequences starting with the splice site “GT”. The 3’ intron sequence means the partial intron sequences ending with the branch point.
(TIF)

S4 Fig Circular RNAs from yeast *dbr1* sequester HYL1 binding to *pri-miR167b*. **(A)** Recombinant proteins of MBP, MBP-HYL1 (only the N-terminal harboring two dsRNA binding domains, D1D2 was fused to MBP), GST, GST-DBR1. (B) R-EMSA to determine if above recombinant proteins binding Biotin-labeled *pri-miR167b*. The arrow indicates the HYL1-*pri-miR167b* complex. (C) R-EMSA to determine if linear RNAs compete for HYL1 binding to *pri-miR167b*. Cold *GAPDH* single-stranded linear RNA transcribed by *in vitro* transcription using the T7 promoter was gradually increased in the reaction, and cold *pri-miR167b* with corresponding concentrations was used as the positive control. The arrow indicates the HYL1-*pri-miR167b* complex. (D) R-EMSA to determine if circular RNAs compete for HYL1 binding, using wild type or a *dbr1* yeast strain. Experiments were performed as Fig 4C, except total RNAs from WT or *dbr1* yeast cells. The arrow indicates the HYL1-*pri-miR167b* complex. (E) Hybridization intensities were quantified and normalized to the controls (lane 1 in D), and are shown in the line graph. Bars represent the average normalized intensity of three biological replicates.
(TIF)

S5 Fig Over-expression of lariat42 caused reduced miRNA accumulation. **(A)** Strategy to over-express lariat42 in tobacco leaves. The intron sequence (indicated as lariat42-OE) was inserted into the split YFP and transiently expressed in tobacco leaves. (B) YFP signals were shown in tobacco leaves infiltrated by the above plasmid. Bright dots indicate YFP signals in nuclei. (C) RT-PCR analysis to detect the expression of lariat42. Total RNA from tobacco leaves infiltrated by the above plasmid or the blank control was used for cDNA synthesis, and lariat42 was amplified to indicate the level of lariat42, *Actin7* as the loading control. (D) Northern blot analysis of miR167 in the control and lariat42-OE. U6 was used as a loading control.
(TIF)

S6 Fig Subcellular localization of DBR1 and DBR1-2 and lariat RNA. (A) Subcellular localization of DBR1 and DBR1-2. 35S::DBR1-YFP and 35S::DBR1-2-YFP were transiently expressed in tobacco leaves and fluorescence signals were observed after 48 hr. (B) Strategy to visualize lariat RNAs in live cells. The MS2 sequence (indicated as stem-loops) was inserted into the lariat41-located intron. A co-expressed GFP-tagged MS2-CP protein was used to visualize lariat41. Grey boxes indicate exons, and lines indicate the intron. (C) HYL1-RFP alone or MS2-CP-GFP and local24-MS2 were transiently expressed in *N*. *benthamiana* leaves. Representative images were shown. (D-E) Three additional representative images of lariat24a (D) and two additional representative images of lariat41 (E) were shown. Scale bars = 10 μm.
(TIF)

S1 Table miRNAs in Col-0 and *dbr1-2*. More than 100 miRNAs with more than 100 RPTM were identified in small RNA sequencing libraries, and are listed with information of their ID, normalized reads (expression, RPTM), and log2 ratio of *dbr1-2*/Col-0, from two biological replicates. The first sheet lists 57 miRNAs that were significantly reduced in the replicate 1 of *dbr1-2*, the second sheet lists 56 miRNAs that were significantly reduced in the replicate 2 of *dbr1-2*, the third sheet lists 54 miRNAs that were commonly reduced in the two replicates of *dbr1-2*, the fourth sheet lists the information of genome structure of 54 commonly reduced miRNAs in *dbr1-2*, and the fifth sheet lists miRNAs co-regulated by both DBR1 and HYL1.
(XLSX)

S2 Table Occupancy of DCL1 and HYL1 at *MIR* genes by RIP-seq analysis. There were 55 and 48 miRNA genes with log2 fold changes of smaller than -1 or larger than 1 identified as deregulated in binding to DCL1 and HYL1, respectively. Most of them, i.e. 47 in the DCL1- and 29 in the HYL1- RIP-seq profiles, had less occupancy of DCL1 and HYL1 in the *dbr1-2* mutant. The genomic location, strand information, binding capacity (RPKM), and log2 ratios of *dbr1-2*/Col-0 from DCL1 and HYL1 RIP-seq were listed in the first and second sheets of the table.
(XLSX)

S3 Table List of introns enriched in *dbr1-2*, as assessed by RNA-seq analysis. The genomic location, strand information, intron ID, and normalized abundance (RPKM) in different RNA-seq libraries, log fold change (logFC), log count per million sequencing reads (logCPM), P-value and False Discovery Rate (FDR) are listed. The intron ID is named according to the accession ID of the corresponding gene, followed by "I" and the intron sequential number. The first sheet lists all 1560 intron RNAs that have significantly different expression levels between Col-0 and *dbr1-2* in RNase R (-) libraries, the second sheet lists the 1534 intron RNAs whose average abundance in *dbr1-2*_RNase R (+) libraries are ≥ 5 from 1560 introns, and the third sheet lists the 360 introns whose average abundance values in Col_RNase R (-) libraries are ≥ 5 RPKM from the 1534 introns on the second sheet.
(XLSX)

S4 Table Primer sequences used in this study. (DOC)
