# Supplementary figures and images for: Intron Lariat RNA Inhibits MicroRNA Biogenesis by Sequestering the Dicing Complex in Arabidopsis
Source: PLoS Genet. 2016 Nov 21;12(11):e1006422. doi: 10.1371/journal.pgen.1006422 (PMC5147768; doi:10.1371/journal.pgen.1006422)

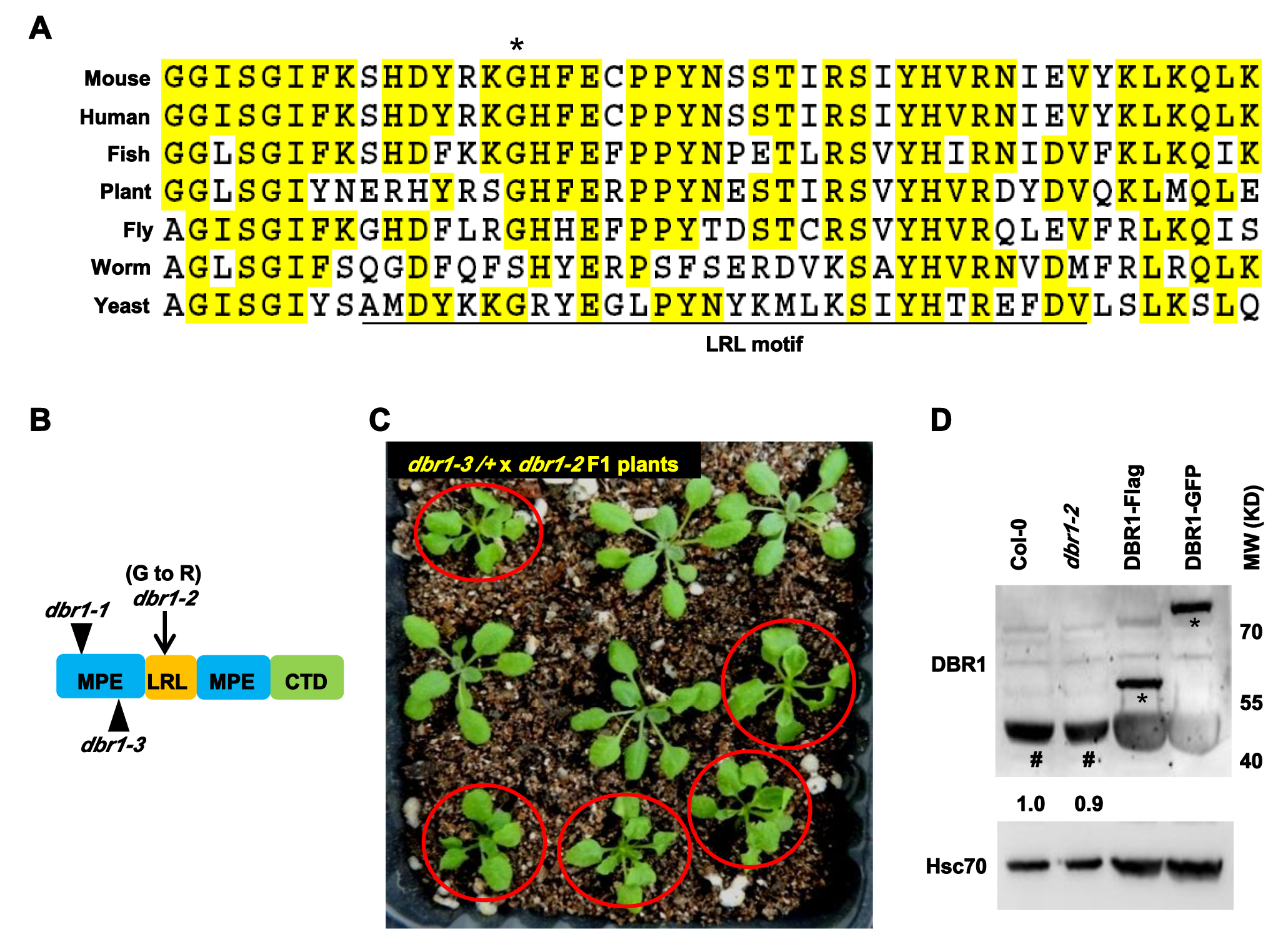

Supplement: S1 Fig — (A) Partial amino acid sequence alignment of DBR1 from various species. DBR1 from Mouse (Mus musculus, NP_113580.2), Human (Homo sapiens, NP_057300.2), Fish (Danio rerio, NP_955947.1), Plant (Arabidopsis thaliana, NP_567881.1), Fly (Drosophila melanogaster, NP_996022.1), Worm (Caenorhabditis elegans, NP_491868.2), and fission yeast (Schizosaccharomyces pombe, NP_593470.2), were aligned using ClustalW. Identical residues are highlighted in yellow. Asterisk indicates the Gly that was mutated to Arg (G135R) in dbr1-2. (B) The protein structure of DBR1. The closed triangle indicates the T-DNA insertion site in dbr1-1 and dbr1-3. The arrow indicates the location of the mutation of glycine to arginine in dbr1-2. MPE indicates the metallophosphoesterase domain, LRL indicates the lariat recognition loop, and CTD indicates a novel C-terminal domain. (C) Allelic analysis of dbr1-2 and dbr1-3. A small field was shown for 3-week-old growing F1 progeny, and dbr1-2-like plants are highlighted by red circles. (D) Western blot to determine DBR1 levels in wild type (Col-0) and dbr1-2 using anti-DBR1 antibody. # indicates the endogenous DBR1 in Col-0 and dbr1-2, and * indicates DBR1-Flag and DBR1-GFP in 35S::DBR1-Flag or 35S::DBR1-GFP transgenic plants, respectively. Notably, the loading amount in Col-0 and dbr1-2 is half of that of two transgenic plants. Hsc70 served as a loading control. (TIF) [file pgen.1006422.s001.tif]

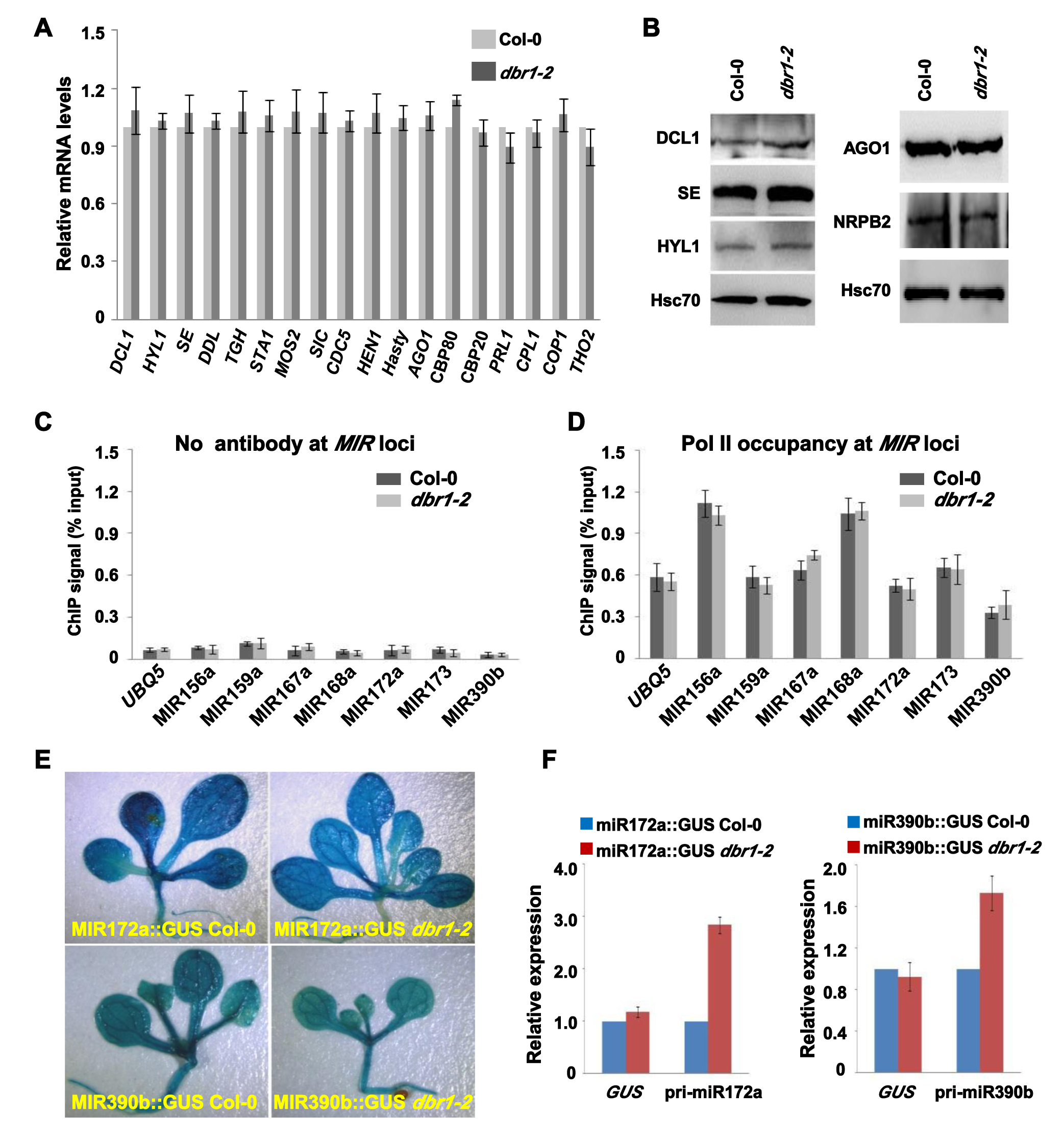

Supplement: S2 Fig — (A) RT-qPCR analysis of mRNA levels of miRNA biogenesis genes. SE was calculated from three biological replicates. (B) Western blot to determine levels of DCL1, HYL1, SE, AGO1, and NRPB2 in Col-0 and dbr1-2. Hsc70 was the loading control. (C) and (D) ChIP performed with Col-0 (dark gray bars) or dbr1-2 (light gray bars) with anti-NRPB2 antibody. SE was calculated from three biological replicates. (E) Representative GUS staining images of MIR172a::GUS and MIR390b::GUS transgenic plants in Col-0 and dbr1-2 backgrounds. (F) RT-qPCR analysis of the transcript levels of GUS, pri-miR172a, and pri-miR390b in the indicated plants. SE was calculated from three biological replicates. (TIF) [file pgen.1006422.s002.tif]

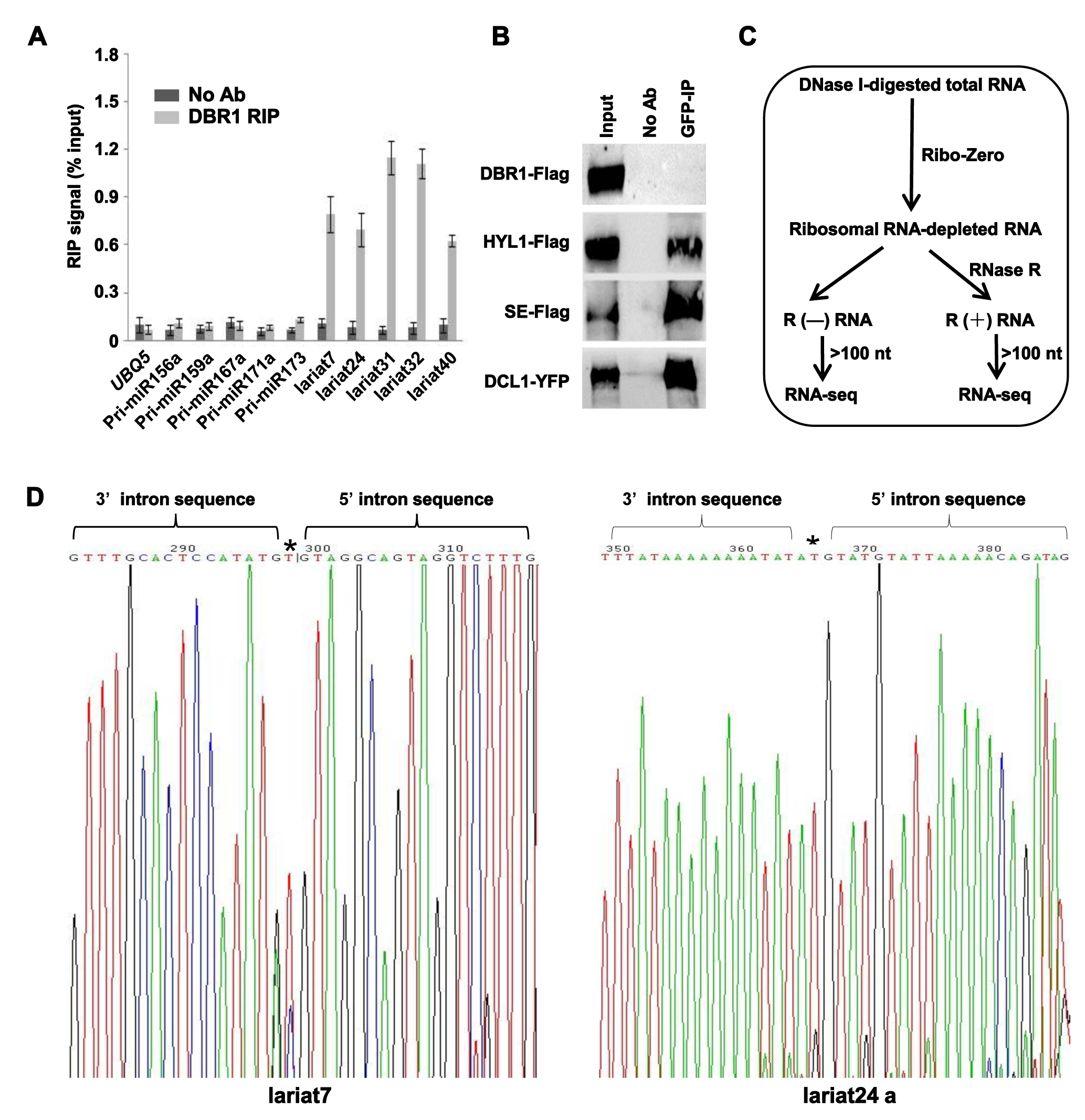

Supplement: S3 Fig — (A) RIP performed with dbr1-2 inflorescences using anti-DBR1 antibody (light gray bars) or no antibody (dark gray bars) as the negative control. RNA was immunoprecipitated from inflorescences of dbr1-2. The amount of pri-miRNAs was determined by RT-qPCR and normalized to the input. UBQ5 was used as a negative control. Lariat RNAs served as positive controls. Error bars show SE calculated from three biological replicates. (B) Co-immunoprecipitation between DCL1 and DBR1, SE, and HYL1. DCL1-YFP was co-expressed with DBR1-Flag, SE-Flag, or HYL1-Flag in N. benthamiana. HYL1-Flag and SE-Flag were used as positive controls. (C) Schematic flow of RNA-seq analysis. 5 μg total RNA were digested by Dnase I, and then RNA were removed ribosomal RNA using Ribo-Zero kit. The half amount of RNAs were treated by RNase R as R(+) RNA to remove linear RNAs, the other half amount of RNAs were used as R(-) RNA. Those RNA fragments with size larger than 100 nt were used for library construction and sequencing. (D) Chromas figures of partial sequences from lariat7 and lariat24a by Sanger sequencing. PCR products of lariat7 and lariat24a from Fig 3C were sequenced using the primer lariat7R and lariat24aR, respectively. The asterisk represents branch points “A” in the DNA sequence, here it was reversed to “T” after cDNA synthesis. The “GT” downstream of the branch point was indicated the 5’ splice site. The 5’ intron sequence means the partial intron sequences starting with the splice site “GT”. The 3’ intron sequence means the partial intron sequences ending with the branch point. (TIF) [file pgen.1006422.s003.tif]

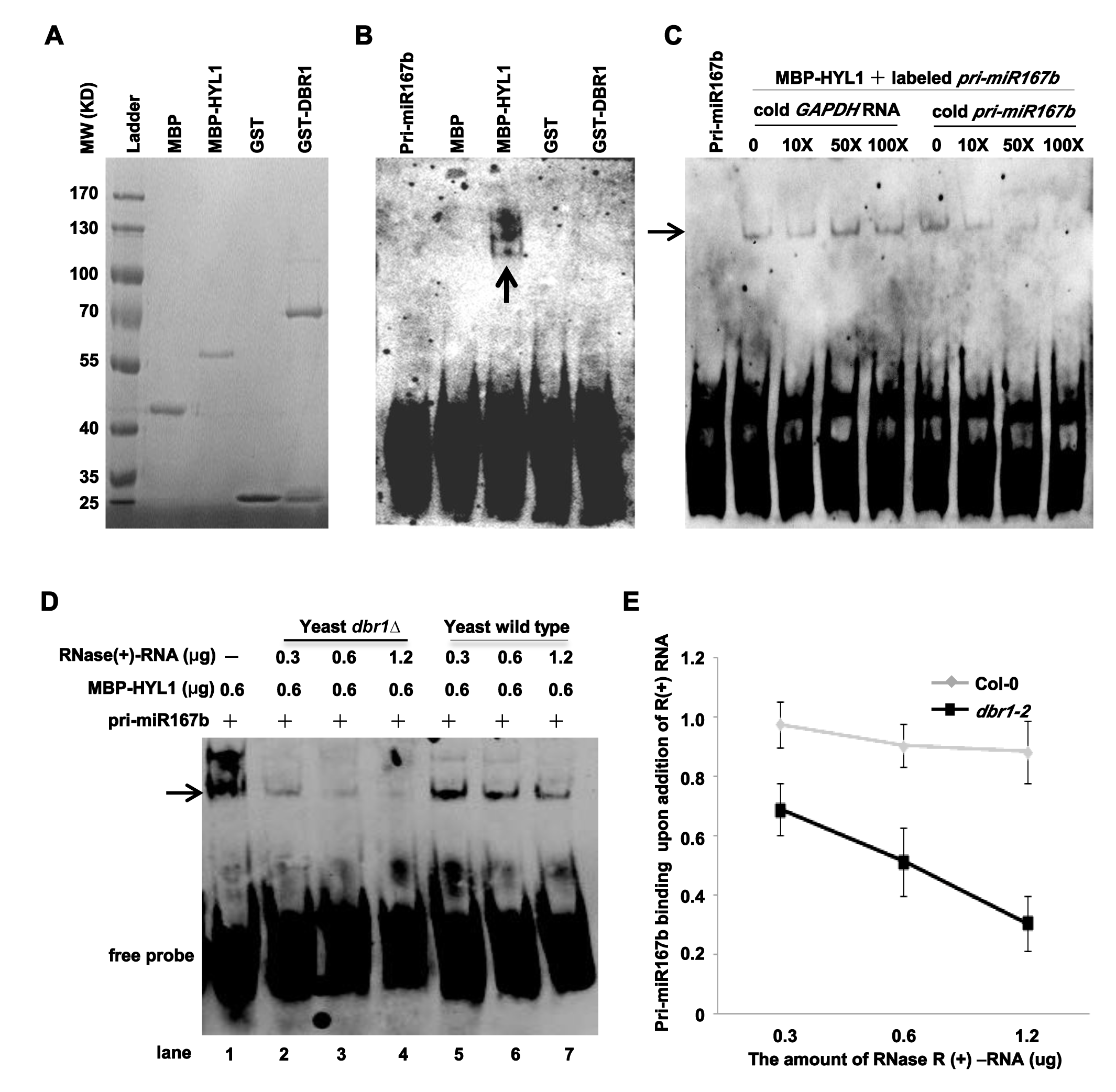

Supplement: S4 Fig — (A) Recombinant proteins of MBP, MBP-HYL1 (only the N-terminal harboring two dsRNA binding domains, D1D2 was fused to MBP), GST, GST-DBR1. (B) R-EMSA to determine if above recombinant proteins binding Biotin-labeled pri-miR167b. The arrow indicates the HYL1-pri-miR167b complex. (C) R-EMSA to determine if linear RNAs compete for HYL1 binding to pri-miR167b. Cold GAPDH single-stranded linear RNA transcribed by in vitro transcription using the T7 promoter was gradually increased in the reaction, and cold pri-miR167b with corresponding concentrations was used as the positive control. The arrow indicates the HYL1-pri-miR167b complex. (D) R-EMSA to determine if circular RNAs compete for HYL1 binding, using wild type or a dbr1 yeast strain. Experiments were performed as Fig 4C, except total RNAs from WT or dbr1 yeast cells. The arrow indicates the HYL1-pri-miR167b complex. (E) Hybridization intensities were quantified and normalized to the controls (lane 1 in D), and are shown in the line graph. Bars represent the average normalized intensity of three biological replicates. (TIF) [file pgen.1006422.s004.tif]

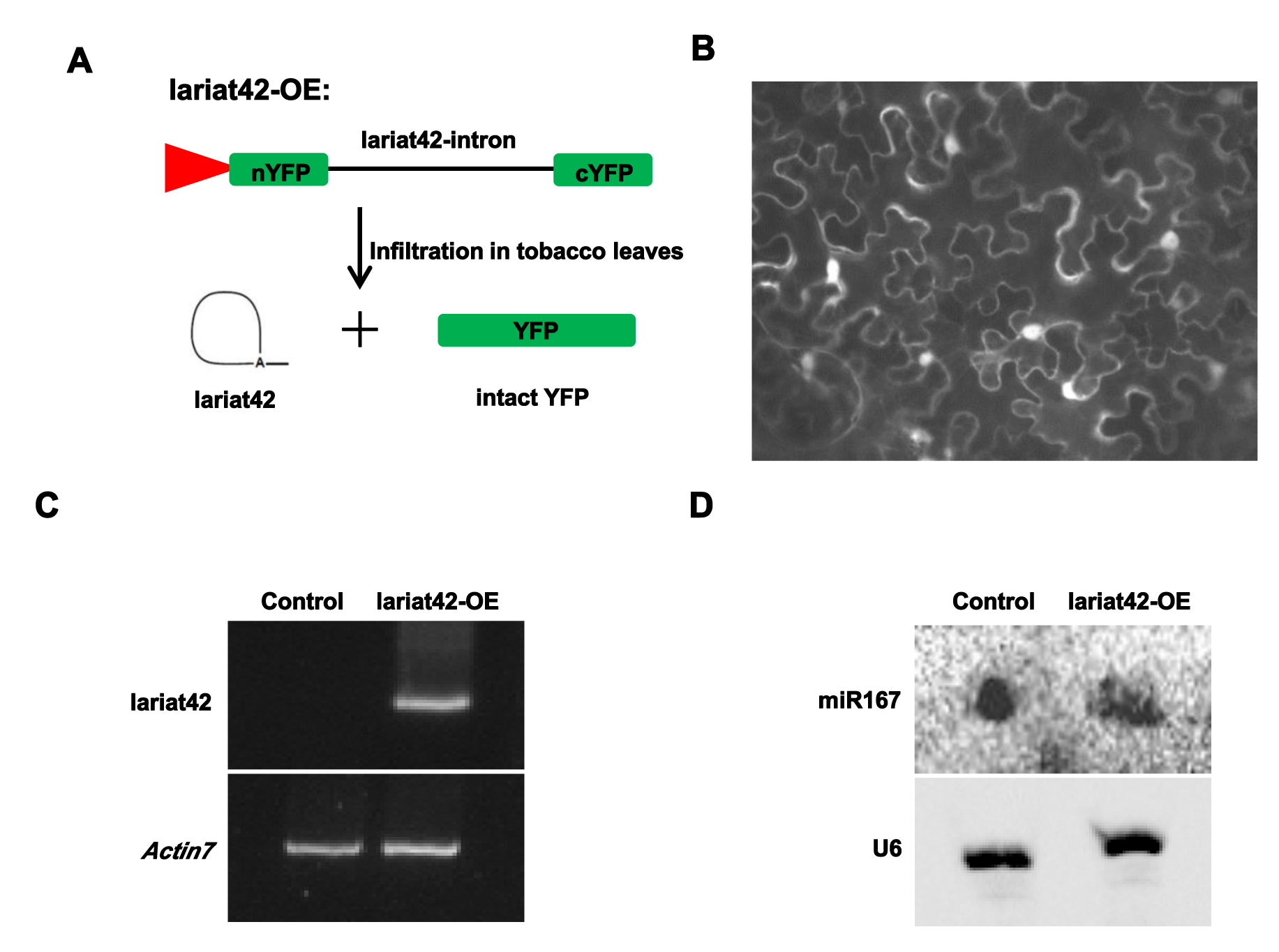

Supplement: S5 Fig — (A) Strategy to over-express lariat42 in tobacco leaves. The intron sequence (indicated as lariat42-OE) was inserted into the split YFP and transiently expressed in tobacco leaves. (B) YFP signals were shown in tobacco leaves infiltrated by the above plasmid. Bright dots indicate YFP signals in nuclei. (C) RT-PCR analysis to detect the expression of lariat42. Total RNA from tobacco leaves infiltrated by the above plasmid or the blank control was used for cDNA synthesis, and lariat42 was amplified to indicate the level of lariat42, Actin7 as the loading control. (D) Northern blot analysis of miR167 in the control and lariat42-OE. U6 was used as a loading control. (TIF) [file pgen.1006422.s005.tif]

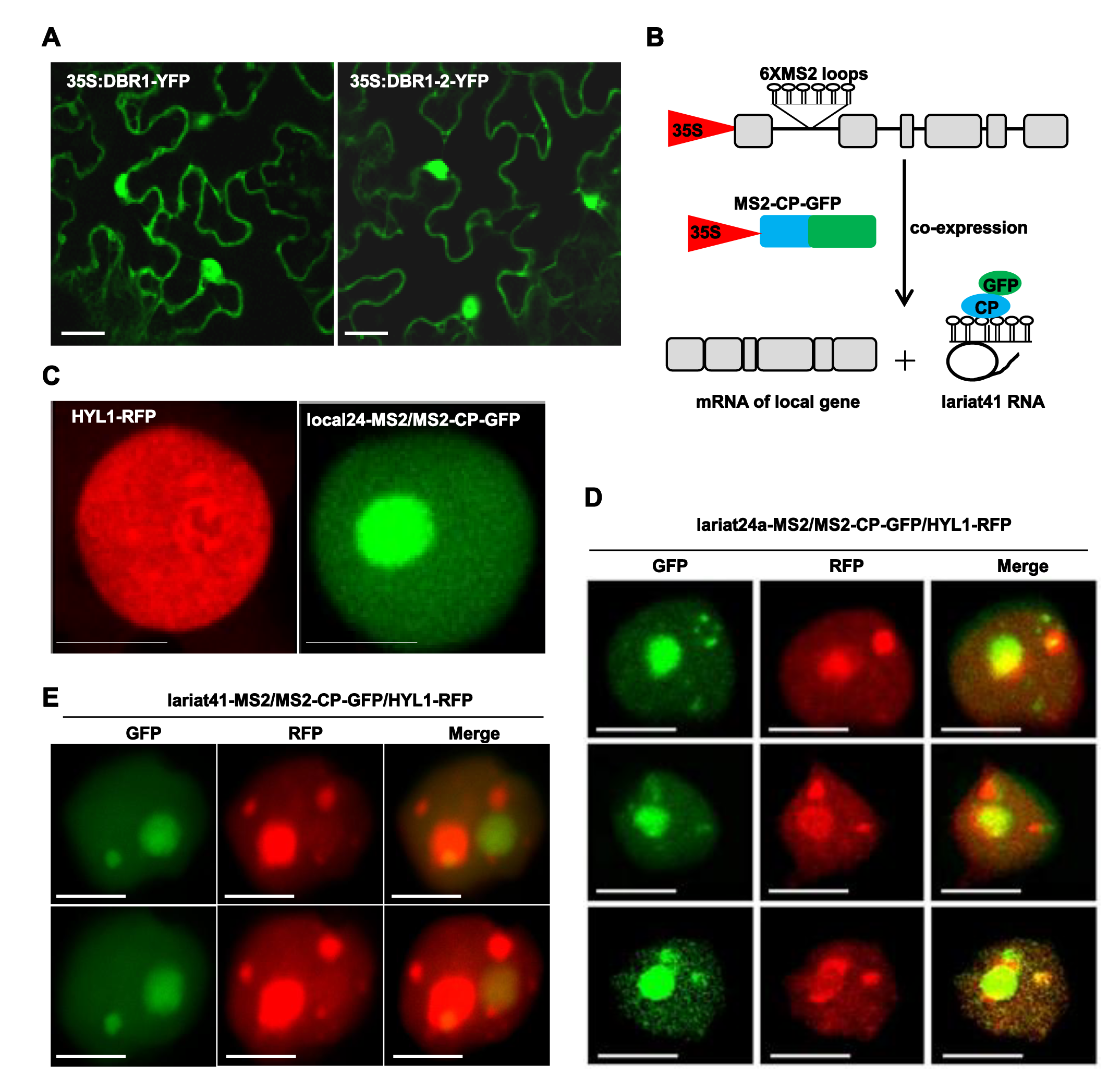

Supplement: S6 Fig — (A) Subcellular localization of DBR1 and DBR1-2. 35S::DBR1-YFP and 35S::DBR1-2-YFP were transiently expressed in tobacco leaves and fluorescence signals were observed after 48 hr. (B) Strategy to visualize lariat RNAs in live cells. The MS2 sequence (indicated as stem-loops) was inserted into the lariat41-located intron. A co-expressed GFP-tagged MS2-CP protein was used to visualize lariat41. Grey boxes indicate exons, and lines indicate the intron. (C) HYL1-RFP alone or MS2-CP-GFP and local24-MS2 were transiently expressed in N. benthamiana leaves. Representative images were shown. (D-E) Three additional representative images of lariat24a (D) and two additional representative images of lariat41 (E) were shown. Scale bars = 10 μm. (TIF) [file pgen.1006422.s006.tif]
