# Supplementary material for: Intron Lariat RNA Inhibits MicroRNA Biogenesis by Sequestering the Dicing Complex in Arabidopsis
Source: PLoS Genet. 2016 Nov 21;12(11):e1006422. doi: 10.1371/journal.pgen.1006422 (PMC5147768; doi:10.1371/journal.pgen.1006422)
Supplement: S4 Table — (DOC) [file pgen.1006422.s010.doc]

**S4 Table. Primer sequences used in this s**tudy.

| **Name** | **Sequence (5’-3’)** | | |
| --- | --- | --- | --- |
| **probes for small RNA gel blot** | | | |
| anti-miR156 | biotin-GTGCTCACTCTCTTCTGTCA | | |
| anti-miR159 | biotin-TAGAGCTCCCTTCAATCCAAA | | |
| anti-miR160 | biotin-TGGCATACAGGGAGCCAGGCA | | |
| anti-miR165 | biotin-GGGGAATGAAGCCTGGTCCGA | | |
| anti-miR167 | biotin-TAGATCATGCTGGCAGCTTCA | | |
| anti-miR168 | biotin-TTCCCGACCTGCACCAAGCGA | | |
| anti-miR171 | biotin-GATATTGGCGCGGCTCAATCA | | |
| anti-miR172 | biotin-ATGCAGCATCATCAAGATTCT | | |
| anti-miR173 | biotin-GTGATTTCTCTCTGCAAGCGAA | | |
| anti-miR319 | biotin-TGGGAGCTCCCTTCAGTCCAA | | |
| anti-miR390 | biotin-GGCGCTATCCCTCCTGAGCTT | | |
| anti-U6 | biotin-TCATCCTTGCGCAGGGGCCA | | |
| **primers for qRT-PCR** | | | |
| N-UBQ5 | GGTGCTAAGAAGAGGAAGAAT | | |
| C-UBQ5 | CTCCTTCTTTCTGGTAAACGT | | |
| Actin7F | TGGCATCAACTTTCTACAA | | |
| Actin7R | CCACCACTAGCACAATGTT | | |
| pri-miR156aF | CAAGAGAAACGCAAAGAAACTGACAG | | |
| pri-miR156aR | AAAGAGATCAGCACCGGAATCTGACAG | | |
| pri-miR158aF | GTGATGACGCCATTGCTCTTT | | |
| pri-miR158aR | TGTGACTTTAGATGCCCTTGTTCA | | |
| pri-miR159aF | GGAGCTCTACTTCCATCGTCA | | |
| pri-miR159aR | CCACGTTCTCATCAAAACTTTC | | |
| pri-miR166aF | GACTCTGGCTCGCTCTATTCA | | |
| pri-miR166aR | TGGTCCGAAGACGCTAAAAC | | |
| pri-miR167aF | GAAGCTGCCAGCATGATCTA | | |
| pri-miR167aR | GGGTTTATAGAAGGGTGCGA | | |
| pri-miR168aF | GCCTTGCATCAACTGAAT | | |
| pri-miR168aR | CAAACAAAAGGAGACTAAAGA | | |
| pri-miR171aF | CCGCGCCAATATCTCAGTA | | |
| pri-miR171aR | TGTCTCCATTTCAACACACACA | | |
| pri-miR172aF | ATCTGTTGATGGACGGTGGT | | |
| pri-miR172aR | AATAGTCGTTGATTGCCGATG | | |
| pri-miR173F | CCCAATACCCTATTTTCCTCT | | |
| pri-miR173R | ATCTCTAACATTAAATCATCA | | |
| pri-miR319aF | GAGATAGAGAGTTGAACAAATTCTTC | | |
| pri-miR319aR | GTATCCATGATAGTTGAGAAATTTGC | | |
| pri-miR390bF | GGAAGATGCATACCTATGGATG | | |
| pri-miR390bR | CATGCAAATAATAAACTTACCC | | |
| MIR156aF | AAAGAGAGGGAGGGAGAGAG | | |
| MIR156aR | CGACAAGAGCCATAAAGAAA | | |
| MIR159aF | AGCTCTActtccatcgtcagat | | |
| MIR159aR | ctctctatctatcatttcttcc | | |
| MIR167aF | cgacccttaaactctccataa | | |
| MIR167aR | ACTTCACCGTAGCAGATCAA | | |
| MIR168aF | CAGTGTGAAAGCGAAAATCT | | |
| MIR168aR | TTGTAATGGGGAAATGAGGT | | |
| MIR171aF | tgctttggtagtagatgaggtt | | |
| MIR171aR | CGTGTGTGGTCAGGTAAGAT | | |
| MIR172aF | TGACTTGTTTTGATTGAAGGA | | |
| MIR172aR | TGTGGGTTTGTCTCGTTTTA | | |
| MIR173F | AAGAGTGGTCGTGTGAGTTG | | |
| MIR173R | GGGTATTGGGCTGAGATTAC | | |
| MIR390bF | CGAACAAAACTCATAAAATCC | | |
| MIR390bR | ATTCCCATACCCACAATAAC | | |
| GUS-F | TAATGTTCTGCGACGCTCACA | | |
| GUS-R | ATACCTGTTCACCGACGACG | | |
| DCL1F | ATTGGGAAAATAGTGACAGC | | |
| DCL1R | TCAAAGGTTGTTGCTTACAG | | |
| HYL1F | CAGTAAAGAGGAGGAAGTCG | | |
| HYL1R | ACACCAGAGGAAACATCAGT | | |
| SEF | AGAAATCAACCTCTTCCTCA | | |
| SER | CGTCTCTTGTAATCCCTACG | | |
| DDLF | GGTCTACTTCACCGTCAGAT | | |
| DDLR | GGTTCTTCCTTTTTCTTCG | | |
| TGHF | CTGCACGTCAAGTATCTGAG | | |
| TGHR | TTCTAGGAAATCGCCAGCTA | | |
| MOS2F | GTGTGAATGAGGTTGCTGAT | | |
| MOS2R | AAGGCTTCACTTTCCTCTCT | | |
| STA1F | GAAGAAGTGTGATCGCGACC | | |
| STA1R | ACACACTTCGCCACAACTTC | | |
| SICF | ATGCAAACTGGGGTGGAAAC | | |
| SICR | CTTGACCTGTTGAGCTGCTG | | |
| CDC5F | GAGGAAGAAGCAAGACAACA | | |
| CDC5R | GTCATCAAGCGGATACTTTG | | |
| PRL1F | CGTCTCAGCCATAAGATGAA | | |
| PRL1R | GGACCAACAACAATAGCACT | | |
| COP1F | TGAAAGCAAAATGGAAGAGA | | |
| COP1R | ATACAAATCGGACAAAGCAA | | |
| CPL1F | ATTATTTGGTGTCGGAGGAT | | |
| CPL1R | GCTGAAGGTTGTATTGCTTG | | |
| TOH2F | ACCTCTACCGCAATACACAA | | |
| TOH2R | GCTTCTTCCACTCTCAACCT | | |
| AGO1F | TGGACCACCGCAGAGACAAT | | |
| AGO1R | CATCATACGCTGGAAGACGACT | | |
| HEN1F | GTTCCATCCTTGAGTTTGAC | | |
| HEN1R | GTTTTCTTCTTGGGTTTCTG | | |
| HastyF | GAGTTTGCTGGTGGAAGTTC | | |
| HastyR | AGCTCTAGATGTACTAGTAG | | |
| CBP80F | AAACCTCTCGGAAGAAGATG | | |
| CBP80R | CCAATCAAAGTCCCATACAA | | |
| CBP20F | AAACGACAAAGAGACGATGA | | |
| CBP20R | ATTGACCATTCAGCAACATT | | |
| local41F | ATGTCTGGAGCATTGAATATG | | |
| local41R | CTTTGCCATGGCAAGCTCCT | | |
| **primers for RT-PCR and qRT-PCR of lariat RNA** | | | |
| lariat7-F | cattatgaacaagacaaagacc | | |
| lariat7-R | cctttgaagcctgtaattcc | | |
| lariat24a-F | ctctacacatatctgtttttaa | | |
| lariat24a-R | gcaattccacacaaatcttttgg | | |
| lariat28-F | catcactttctagggtttccg | | |
| lariat28-R | ctttggtggcaatgagagtg | | |
| lariat31-F | cgagtagaagtagcagaaatc | | |
| lariat31-R | ctgaattcgtctttgcgttc | | |
| lariat32-F | caaaaagtaagagatgagaag | | |
| lariat32-R | ccttctcacatgatgatattc | | |
| lariat35-F | gaaaaggctgatgatagctatcc | | |
| lariat35-R | gttggtatgcattcattctgg | | |
| lariat36-F | gttcgaattacaaaaagacg | | |
| lariat36-R | gatctttcaggtcctgtaatc | | |
| lariat39-F | caccaagtaggcctaaagacc | | |
| lariat39-R | catttctgcagtactgttttac | | |
| lariat40-F | ccatgagatgagaatgcatag | | |
| lariat40-R | gcagagagaaaaaccttaaaag | | |
| lariat41-F | ccaaatcatacacatagaag | | |
| lariat41-R | gaacatgcttctttctagttc | | |
| lariat42-F | gtggagactaaagactcgtg | | |
| lariat42-R | ccaagcacttgcatcacaatcca | | |
| linear7-F | CACCTCTTGGTCCTACCTTT | | |
| linear7-R | TCACCAAGTAATCTCCGAAA | | |
| linear24a-F | TAGAAAAGTCCGCCGTTGT | | |
| linear24a-R | GGGTTTATCCTCCTCTGCT | | |
| linear36-F | GTGTTGTGAACGGAGTTGAT | | |
| linear36-R | TCCACGGTTTCTGTATGTTT | | |
| linear40-F | CACCTCTTGGTCCTACCTTT | | |
| linear40-R | TACGGGTTTGTAATGTGCTC | | |
| **primers for vector construction** | | | |
| DBR1F1 | | | CACCgacgagggaggacgattggag |
| DBR1R1 | | | TGCATCGTCTCTTGTATGATCA |
| DBR1F2 | | | CGCggtaccATGAAGATTGCAATTGAAGGTTG |
| DBR1R2 | | | CGCggatccTGCATCGTCTCTTGTATGATCATC |
| DBR1F3 | | | CGCggatccATGAAGATTGCAATTGAAGGTTG |
| DCL1F1 | | | caccATGGTAATGGAGGATGAGCCTA |
| DCL1R1 | | | AGAAAAAGTTTTATTTAAAAGCTC |
| HYL1F1 | | | CACC atgacctccactgatgtttcc |
| HYL1R1 | | | TGCGTGGCTTGCTTCTGTCT |
| SEF1 | | | ctcGGTACCatggccgatgttaatcttcct |
| SER1 | | | cgcGGATCCcaagctcctgtaatcaataacg |
| lariat41F2 | | | ATGTCTGGAGCATTGAATATG |
| lariat41R2 | | | AGAGGTGTTCATGGCATCAGC |
| lariat42F2 | | | cgcCTCGAGgtaagatttcttgtatataac |
| lariat42R2 | | | cgcCTCGAGctgcaatacgacataatcac |
| gL24F | | | caccATGGGAGAGGTTCAAGAAAATCC |
| gL24R | | | CATTACAGTACATGCATTTGG |
| gL41F | | | ATGTCTGGAGCATTGAATATG |
| gL41R | | | AGAGGTGTTCATGGCATCAGC |
| 6XMS2loops-F1 | | | gcgGAAcgcgTTctagctgaggatcctaaggtacctaattgcctag |
| 6XMS2loops-R1 | | | gcgGAAcgcgTTCtacccggggatctaatgaacccgggaatactgcag |
| 6XMS2loops-F2 | | | gcgGAGCTCatcctaaggtacctaattgccta |
| 6XMS2loops-R2 | | | gcgGAGCTCggatctaatgaacccgggaatact |
| miR163-MS2-F | | | CACCacagttctcatcaaatatttga |
| miR163-MS2-F | | | gatcataaatatattttgtgtac |
| **primers for *in vitro* transcription** | | | |
| T7primiR167bF | | TAATACGACTCACTATAGGatttctccacttcttgagcttcc | |
| primiR167bR | | AGTCAACTGTGTGCGTTCGGGACT | |
| T7GAPDH-F | | TAATACGACTCACTATAGGgagaatggccttatcttctctcct | |
| GAPDH-R | | caaacgcaagagtgagaaacg | |
